# Nucleophilic displacement reactions of 5′-derivatised nucleosides in a vibration ball mill

**DOI:** 10.3762/bjoc.13.11

**Published:** 2017-01-13

**Authors:** Olga Eguaogie, Patrick F Conlon, Francesco Ravalico, Jamie S T Sweet, Thomas B Elder, Louis P Conway, Marc E Lennon, David R W Hodgson, Joseph S Vyle

**Affiliations:** 1 School of Chemistry and Chemical Engineering, Queen’s University Belfast, David Keir Building, Stranmillis Road, Belfast BT9 5AG, UK; 2 Durham University, Department of Chemistry, Lower Mountjoy, Stockton Road, Durham DH1 3LE, UK

**Keywords:** ball mill, chalcogen, mechanochemistry, nucleophilic substitution, nucleoside

## Abstract

Vibration ball-milling in a zirconia-lined vessel afforded clean and quantitative nucleophilic displacement reactions between 4-methoxybenzylthiolate salts and nucleoside 5′-halides or 5′-tosylates in five to 60 minutes. Under these conditions, commonly-encountered nucleoside cyclisation byproducts (especially of purine nucleosides) were not observed. Liquid-assisted grinding of the same 5'-iodide and 5′-tosylate substrates with potassium selenocyanate in the presence of DMF produced the corresponding 5′-selenocyanates in variable yields over the course of between one and eleven hours thereby avoiding the preparation and use of hygroscopic tetrabutylammonium salts.

## Introduction

Nucleophilic displacement reactions of nucleoside derivatives are well established owing to the accessibility of precursor sulfonate esters and halides [1]. Displacements with a diverse variety of both hard and soft nucleophiles have enabled the construction of natural products [2–3] and their analogues [4–6]. Typically, such reactions require the use of high-boiling, dipolar aprotic solvents and anionic nucleophiles under anhydrous conditions at elevated temperatures (up to 150 °C). Competing intramolecular cyclisation reactions between both purine and pyrimidine nucleobases and (especially) the 5′-position of the (deoxy)ribofuranoside are frequently observed during both activation [7] and subsequent displacement reactions [8–9]. This competition confounds the kind of systematic analysis developed recently by Hale and co-workers for sulfonate displacement from furanosides [10] and convoluted optimisations of reaction conditions and nucleoside substrate (e.g., the leaving group [11] or nucleobase protection [12]) are therefore often required.

We anticipated that some of these issues could be ameliorated through mechanochemical mixing of reactants and substrates, with diverse solubility profiles, using minimal or no added liquid [13–15]. Recently, Jicsinszky et al. described using a planetary ball mill to perform substitution reactions of 6^I^-*O*-(*p*-toluenesulfonyl)-β-cyclodextrin (Ts-β-CD) with azide, halide or thiolate nucleophiles and thereby avoided intramolecular cyclisation (commonly found under solution-phase conditions) [16].

Following Sikchi and Hultin’s original description of nucleoside derivatisation in a low-energy, planetary ball mill [17], commercial, higher energy vibration mills have been used to facilitate established [18–21] and unprecedented [22] nucleoside transformations. Remarkably, S_N_2 ball-milling chemistry on nucleoside substrates has not, to the authors’ knowledge, been demonstrated, despite reports of similar chemistry on glycoside derivatives [16,23] and α-amino acid analogues [24].

We describe here the efficient displacement of 5'-chloride, iodide or tosylate leaving groups from nucleosides by 4-methoxybenzylthiolate using vibration ball milling (VBM) where competing intramolecular cyclisation reactions are completely avoided. We further demonstrate the application of liquid-assisted grinding to related displacements using potassium selenocyanate.

## Results and Discussion

**Scheme 1:**
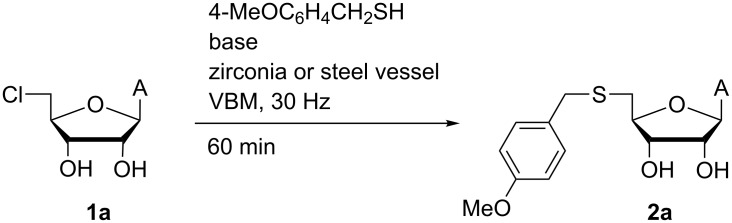
Model reactions of 5′-chloro-5′-deoxyadenosine (**1a**) with 4-methoxybenzylthiolate salts used to optimise VBM conditions.

We initially examined VBM-facilitated reactions of 5′-chloro-5′-deoxyadenosine (CldA, **1a**) with 4-methoxybenzylthiol (MobSH) to generate the corresponding 5′-thioether (MobSdA, **2a**) as a model system (Scheme 1). Despite the known reactions of **1a** with aryl and alkyl chalcogenate salts [6], there is only a single (patent) report of CldA substitution using the Mob-thiolate anion (requiring 12 hours at up to 55 °C to achieve 68% yield) [25].

Optimisation of VBM conditions was performed over several phases using 25 mL vessels and 15 mm diameter balls (both with either steel or zirconia components in contact with the reaction mixtures) operating at 30 Hz (Table 1). Divakar and Reese described the efficient synthesis of 2′-*S*-Mob-2′-thiouridine under solvothermal conditions using large excesses of both MobSH (3 equiv) and 1,1,3,3-tetramethylguanidine (TMG, 5 equiv) [26] and applying this reagent stoichiometry to the ball mill reaction gave complete and clean conversion of CldA (**1a**) into MobSdA (**2a**) monitored by TLC within 1 h in a previously unused steel vessel (Table 1, entry 1).

**Table 1:** Optimisation of VBM conditions for the reaction of 5′-chloro-5′-deoxyadenosine (**1a**) with MobSH. All reactions were carried out at 30 Hz for 60 minutes (unless otherwise stated).^a^

Entry	Base (equiv)	Scale [mmol]	Jar/Ball	Equiv of MobSH	Isolation method^b^	Yield (%)^c^

1	TMG (5.0)	1.05	steel	3.0	A	68
2	TMG (5.0)	0.35–1.0	steel	2.5	A	19–52^d^
3	TMG (5.0)	0.35	steel	1.5	–	(70)^e^
4	–	0.35	steel	3.0	–	–
5	Et_3_N (5.0)	0.35	steel	3.0	–	–
6	Na_2_CO_3_ (7.0)	0.35	steel	3.0	–	–
7	NaOH (7.0)	0.35	steel	3.0	–	(100)^e^
8	TMG (5.0)	1.05	ZrO_2_	3.0	A	66
9	TMG (5.0)	1.0	ZrO_2_	3.0	B	87
10	TMG (5.0)	1.0	ZrO_2_	3.0	C	77

^a^Mob: 4-methoxybenzyl; TMG: 1,1,3,3-tetramethylguanidine; ^b^A: crystallisation; B: chromatography; C: trituration (see Supporting Information File 1 for details). ^c^Isolated. ^d^Complete reaction required up to 105 minutes (for 1.0 mmol scale) in service-aged vessels. ^e^Estimated conversion by TLC.

The efficiency of chloride displacement from CldA (**1a**) was compromised in the presence of fewer equivalents of thiol (Table 1, entries 2 and 3). Furthermore, in the absence of base (Table 1, entry 4) or using different bases (Table 1, entries 5 and 6), no such reaction was observed and CldA remained untransformed. In the presence of sodium hydroxide, an efficient reaction was observed by TLC (Table 1, entry 7), however, the intractability of the resultant reaction mixtures and higher corrosion levels led to further studies being exclusively restricted to TMG.

During multiple iterations of the reaction using steel components, highly variable rates and yields were found (Table 1, entry 2). We correlated these yields, the degree of discolouration of the reaction mixtures and their levels of contamination by metallic, particulate matter with the service-age of the steel vessel. These observations appear consistent with previously described chemical (leaching) and physical (pitting and exfoliation) corrosion of steel vessels in the presence of sulfur-containing materials [27] although Lamaty and co-workers described contrary results [28]. Specifically, in this latter case, a comprehensive survey of metal concentrations within reaction mixtures from both planetary and vibration ball mills was performed and showed lower contamination in the service-aged steel vessel although it should be noted that the steel vessels were from different sources. However, in agreement with this work, we, too were able to remove metallic impurities by extracting the reaction mixture into an organic solvent, filtering the suspension (if solid particulates were present) and crystallising the residue (isolation procedure A). We thus moved to using zirconia components, where no solid impurities were observed, and the yield of MobSdA (**2a**) was comparable (Table 1, entry 8) despite the lower density of ZrO_2_ (6.06 g cm^−3^) compared with steel (7.74 g cm^−3^).

Using zirconia components, reaction mixtures were viscous pale yellow-brown liquids which could be readily removed from the vessel by rinsing with methanol and water and subsequently purified by chromatography (Table 1, entry 9) or trituration (Table 1, entry 10).

**Scheme 2:**
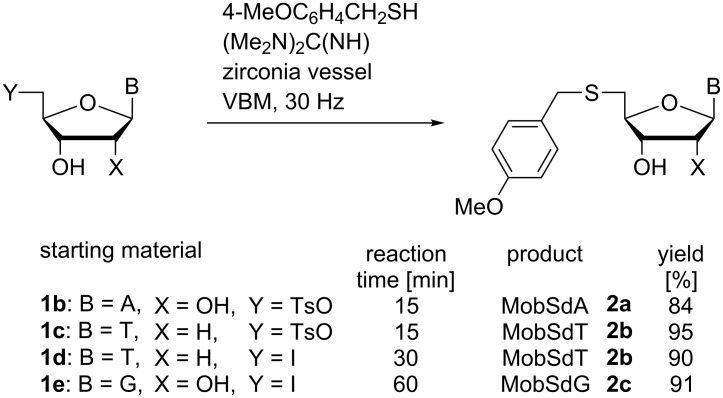
Thiolate displacement reactions of 5′-derivatised nucleosides using VBM.

Using these optimised conditions, we explored displacement reactions of a variety of 5′-tosyl- and 5′-iodo-nucleoside derivatives and in all cases, we observed rapid, complete and clean conversions, with very good to excellent isolated yields (Scheme 2).

5′-Tosyladenosine (TsA, **1b**) was transformed more efficiently than the corresponding halonucleoside and no additional handling was required during work-up of the thioether product (**2a**). Likewise, 5′-tosylthymidine (TsT, **1c**) was rapidly consumed. Only in the case of IdT (**1d**) were any (minor) impurities observed when all reagents were added to the vessel prior to commencing VBM. These impurities were avoided by pre-milling the substrate with MobSH prior to addition of the base (during the initial milling, no chemical reaction was observed). In addition, the work-up developed for MobSdA (**2a**) had to be modified in response to the aqueous solubility of nucleosides containing lactam groups which are deprotonated under basic conditions, i.e., MobSdT (**2b**) and MobSdG (**2c**) [29–30]. In these cases, reaction mixtures were removed from the vessels using water and ethyl acetate. Careful acidification of the aqueous phase enabled MobSdT (**2b**) to be extracted into ethyl acetate prior to trituration. Following the same procedure, crystallisation of MobSdG (**2c**) was induced during acidification. The minimum time required for complete consumption of TsT (**1c**) was found to be five minutes and the isolation procedure could be further expedited (at some cost to the recovery of **2b**) by removing the reaction mixture from the jar in acetonitrile, precipitation from water under acid conditions and triturating the solids (see Supporting Information File 1 for details). We note that only **2b** has been described previously in peer-reviewed literature although without details of its preparation [31].

Following reports of halogen exchange reactions using both stoichiometric [16,32–33] and catalytic [24] halide ions using ball milling, we investigated the capacity of iodide to accelerate the rate of mechanochemically-activated displacement from CldA (**1a**). However, we observed no change in the reaction rate following addition of NaI (500, 50 or 5 mol %) or TBAI (50 or 5 mol %).

**Scheme 3:**
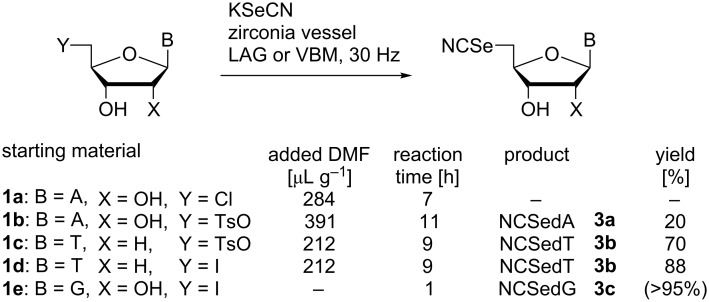
Selenocyanate displacement reactions of 5′-derivatised nucleosides using liquid-assisted grinding (LAG) or VBM.

Following the successful application of VBM using a thiolate nucleophile, we were prompted to explore related reactions of alkylselenols. However, the sensitivity of such compounds towards aerial oxidation meant that, in our hands, an efficient and reproducible reaction of the nucleoside substrates with MobSeH was not achieved in a ball mill. We therefore moved to investigate displacements using potassium selenocyanate which is commercially available, less sensitive to oxidation, and is known to react selectively at selenium in reactions with alkyl halides [34]. We confirmed the efficiency of such VBM-promoted chemistry using benzyl bromide and MobCl as model substrates in the presence of KSeCN (1.1 equiv). Both reactions proceeded cleanly to completion within 60 minutes at 30 Hz and BnSeCN and MobSeCN were isolated in yields of 97% and 99%, respectively (see Supporting Information File 1). We observed more variable outcomes when using KSeCN on nucleoside substrates (Scheme 3).

In contrast with the rapid and clean displacement reactions of 5′-adenosine derivatives in the presence of a thiolate nucleophile, we found CldA (**1a**) remained untransformed during (dry) VBM or liquid-assisted grinding (LAG) in the presence of DMF with excess KSeCN (3 equiv). As found previously, the addition of catalytic TBAI (vide supra) did not alter this outcome nor did the use of a larger excess (5 equiv) of selenocyanate. The addition of DMF to the reaction of TsA (**1b**) with KSeCN suppressed the formation of a polar side-product (putative *N*^3^,5′-cyclonucleoside – also observed as the sole product using LAG in the absence of selenocyanate) sufficiently for the corresponding 5′-selenocyanate (**3a**) to be isolated in poor yield after LAG for 11 hours. Suspending the reaction mixture in methanol and quenching excess selenocyanate with benzyl bromide enabled pure **3a** to be isolated following extensive purification.

In contrast, LAG of TsT (**1c**) with DMF for 9 h achieved 90% conversion (by TLC) to a single product. Likewise, efficient (ca. 95%) conversions were observed with IdT (**1d**) and IdG (**1e**) although maximal conversion of IdG was achieved within one hour in the absence of added liquid (the reaction was inhibited in the presence of DMF). During these studies, LAG of both purine and pyrimidine substrates in the presence of ethyl acetate or hexane was attempted but in all cases the levels of transformation were reduced compared with DMF. Extraction of NCSedT (**3b**) from the reaction vessels in methanol and quenching excess inorganic selenocyanate with benzyl bromide enabled the product to be purified by silica gel column chromatography. In the absence of the quench step, contamination of the product by varying levels of inorganic selenocyanate was found.

Although the reaction mixture derived from IdG (**1e**) could be extracted directly as a solid or into DMSO-*d*_6_ from which mass spectrometry, ^77^Se and ^1^H NMR data consistent with the formation of NCSedG (**3c**) in high yield was inferred, in our hands, this product could not be readily purified. In particular, during trituration with solvents in which excess KSeCN was soluble (e.g., acetone), a loss in the ^77^Se NMR signal associated with **3c** (δ_Se_ 186.8) was observed concomitant with an enhancement in that of the putative corresponding diselenide (δ_Se_ 283.1).

Belostotskii et al. have previously reported the synthesis of 5'-selenocyanate-nucleoside derivatives [35]; however, our approach offers the convenience of an 'off-the-shelf' reagent rather than requiring the synthesis of a tetrabutylammonium (TBA) salt. Furthermore, KSeCN does not suffer from the formation of hydrates that frequently modulate the efficiency of TBA salts [36–37] and the inconsistent reaction outcomes associated with the hygroscopic nature of such salts is particularly poignant in the light of the reported solvent-dependency also observed with KSeCN under Finkelstein conditions [38]. Finally, our approach also offers the convenience of employing tosylate and halide precursors rather than relying on the esoteric tresylate leaving group system.

## Supporting Information

File 1 Experimental part.
