# Supplementary material for: Nucleophilic displacement reactions of 5′-derivatised nucleosides in a vibration ball mill
Source: Beilstein J Org Chem. 2017 Jan 13;13:87–92. doi: 10.3762/bjoc.13.11 (PMC5238552; doi:10.3762/bjoc.13.11)
Supplement: File 1 — Experimental part. [file Beilstein_J_Org_Chem-13-87-s001.pdf]

Supporting Information File

for

Nucleophilic displacement reactions of 5′-derivatised nucleosides in a vibration ball mill

Olga Eguaogie[1], Patrick F. Conlon[1], Francesco Ravalico[1], Jamie S. T. Sweet[1], Thomas B. Elder[1], Louis P. Conway[2], Marc E. Lennon[1], David R. W. Hodgson[2], and Joseph S. Vyle[*1]

Address: [1]School of Chemistry and Chemical Engineering, Queen’s University Belfast, David Keir Building, Stranmillis Road, Belfast BT9 5AG, UK and [2]Durham University, Department of Chemistry, Lower Mountjoy, Stockton Road, Durham DH1 3LE, UK

Email: Joseph S. Vyle* - j.vyle@qub.ac.uk

* Corresponding author

Experimental part

CONTENTS

General information S2

Experimental procedures and material characterisation

5′-*S*-(4-Methoxybenzyl)-5′-thioadenosine (**2a**) - MobSdA..S4

5′-*S*-(4-Methoxybenzyl)-5′-thiothymidine (**2b**) - MobSdT..S5

5′-*S*-(4-Methoxybenzyl)-5′-thioguanosine (**2c**) - MobSdG S7

5′-Deoxyadenosinyl-5′-selenocyanate (**3a**) - NCSedA S8

5′-Deoxythymidinyl-5′-selenocyanate (**3b**) - NCSedT..S9

5′-Deoxyguanosinyl-5′-selenocyanate (**3c**) - NCSedG S10

Benzylselenocyanate - BnSeCN S11

4-Methoxybenzylselenocyanate - MobSeCN S12

Analytical data

5′-*S*-(4-Methoxybenzyl)-5′-thioadenosine (**2a**) - MobSdA S13
5′-*S*-(4-Methoxybenzyl)-5′-thiothymidine (**2b**) - MobSdT S18
5′-*S*-(4-Methoxybenzyl)-5′-thioguanosine (**2c**) - MobSdG S23
5′-Deoxyadenosinyl-5′-selenocyanate (**3a**) - NCSedA S28
5′-Deoxythymidinyl-5′-selenocyanate (**3b**) - NCSedT S33
5′-Deoxyguanosinyl-5′-selenocyanate (**3c**) - NCSedG S38
Benzylselenocyanate - BnSeCN S42
4-Methoxybenzylselenocyanate - MobSeCN S45

General information

Nucleoside substrates were either used as supplied (5′-*O*-(*p*-toluenesulfonyl)-adenosine Carbosynth NT07155) or prepared according to literature methods.[1-5] Reagents 4-methoxybenzylthiol (90%, Aldrich 113158), 1,1,3,3-tetramethylguanidine (Aldrich 241768), 4-methoxybenzyl chloride (98%, Aldrich 270245), benzyl bromide (98%, Aldrich B17905) and potassium selenocyanate (Aldrich 216186 or Acros Organics 201980100) were used as supplied. Organic solvents were purchased from commercial suppliers and used without further purification.

Aqueous solutions were prepared using H_2O purified to 18.2 MΩ by reverse osmosis (Barnstead NANOpure Diamond water purification system).

Davisil silica gel (60 Å) was oven dried at ca. 120 °C overnight prior to use for flash chromatography. TLC was performed using Merck Kieselgel 60 F254 plates and materials visualised by UV irradiation (254 nm), and/or 3% (w/v) phenol in 95:5 (v/v) ethanol/conc. H_2SO_4 (for nucleosides); KSeCN was further visualised by extended irradiation of the plate at 254 nm which deposited red elemental selenium. Where appropriate, the plates were subsequently heated at high temperature (ca.100–200 °C).

All ball mill reactions were performed using a Retsch Mixer Mill MM 400 vibrating at 30 Hz with either a 15 mm stainless steel ball (13.67 g) in a stainless steel vessel (25 mL internal volume) or a 15 mm zirconia ball (10.70 g) in a zirconia-lined vessel (25 mL internal volume) according to the conditions described below.

^{1}H or proton-decoupled ^{13}C NMR spectra were recorded on a Bruker Avance III-400 MHz or Bruker Ascend-600 MHz at 300 K with residual protonated solvent as internal

standard. ^{77}Se NMR (proton-coupled) were recorded on a Bruker Ascend-600 MHz at 300 K using an insert containing 0.25 M KSeCN in D_2O as external standard (−329.00).[6]

Mass spectra were recorded using a VG Quattro II Triple Quadrupole Mass Spectrometer (Electrospray). Mass spectrometry was performed by Analytical Services and Environmental Projects (ASEP) at Queen's University Belfast.

References

1. Anglin, J. L.; Deng, L. S.; Yao, Y.; Cai, G. B.; Liu, Z.; Jiang, H.; Cheng, G.; Chen, P. H.; Dong, S.; Song, Y. C. *J. Med. Chem.* **2012**, *55*, 8066-8074.
2. Dean, D. K. *Synth. Commun.* **2002**, *32*, 1517-1521.
3. Liu, J.; Zhang, J.; Chen, Y.; Wen, X.; Wang, H.; Lu, Y. *Chin. J. Med. Chem.* **2002**, *12*, 50-51.
4. Conway, L. P.; Mikkola, S.; O'Donoghue, A. C.; Hodgson, D. R. W. *Org. Biomol. Chem.* **2016**, *14*, 7361-7367.
5. Williams, D. M.; Harris, V. H.: Phosphorus methods in nucleotide chemistry. In *Organophosphorus Reagents: a Practical Approach in Chemistry*; Murphy, P. J., Ed.; Oxford University Press: Oxford, 2004; pp 237–272.
6. Verlinden, K.; Buhl, H.; Frank, W.; Ganter, C. *Eur. J. Inorg. Chem.* **2015**, 2416-2425.

Experimental procedures and material characterisation

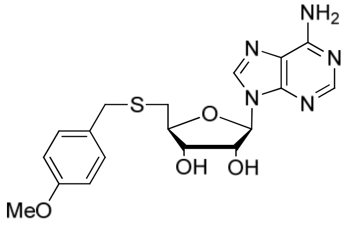

5′-*S*-(4-Methoxybenzyl)-5′-thioadenosine (**2a**) – MobSdA

METHOD A (crystallisation). Under a gentle stream of argon, either a virgin stainless steel jar or a zirconia-lined jar was sequentially charged, in quick succession with 5′-chloro-5′-deoxyadenosine (**1a**, 300 mg, 1.05 mmol), 4-methoxybenzylthiol (488 µL, 3.15 mmol, 3 equiv), 1,1,3,3-tetramethylguanidine (659 µL, 5.25 mmol, 5 equiv) and a 15 mm diameter steel or zirconia ball. The jar was then sealed and vibrated at 30 Hz for one hour and allowed to cool to ambient temperature. The crude reaction mixture was then extracted from the vessel in MeOH (50 mL) and the solvents were removed in vacuo.

Pure MobSdA (**2a**) was subsequently isolated following crystallisation from 6.5:1 (v/v) water/ethanol and washing the white crystals with 1:1 (v/v) *n*-hexane/diethyl ether (20 mL).

Yield: 289 mg (68%) - steel; 280 mg (66%) - zirconia.

METHOD B (chromatography). Under a gentle stream of argon, a zirconia-lined jar was sequentially charged, in quick succession, with 5′-chloro-5′-deoxyadenosine (**1a**) (286 mg, 1.0 mmol) 1,1,3,3-tetramethylguanidine (627 µL, 5.0 mmol, 5 equiv), 4-methoxybenzylthiol (418 µL, 3.0 mmol, 3 equiv) and a 15 mm diameter zirconia ball. The jar was then sealed and vibrated at 30 Hz for one hour and allowed to cool to ambient temperature. The crude reaction mixture was extracted from the vessel in MeOH (2 × 25 mL) and water (10 mL) and the solvents were removed in vacuo. The residues were adsorbed onto silica and subjected to silica gel column chromatography using isocratic elution with 8:92 (v/v) MeOH/DCM. Appropriate fractions containing pure **2a** as a white amorphous solid were pooled and dried in vacuo.

Yield: 350 mg (87%).

METHOD C (trituration). Under a gentle stream of argon, a zirconia-lined jar was sequentially charged with either 5′-chloro-5′-deoxyadenosine (**1a**, 286 mg, 1.0 mmol) or 5′-*O*-(*p*-toluenesulfonyl)adenosine (**1b**, 421 mg, 1.0 mmol). Subsequently, in quick succession, 1,1,3,3-tetramethylguanidine (627 µL, 5.0 mmol, 5 equiv), 4-methoxybenzylthiol (418 µL, 3.0 mmol, 3 equiv) and a 15 mm diameter zirconia ball were added to the jar which was then sealed and vibrated at 30 Hz for either

one hour (**1a**) or 15 minutes (**1b**) and allowed to cool to ambient temperature. The crude reaction mixtures were then extracted from the vessel in MeOH (2 × 25 mL) and water (10 mL) and the solution was reduced in vacuo. The residues were triturated with water (100 mL) under sonication, the suspension was cooled on ice and the solids were isolated by filtration. The solids were further washed with ice-cold water (50 mL), dried in vacuo over P_2O_5 and triturated with 5:1 (v/v) diethyl ether/*n*-hexane (60 mL) under sonication. The suspension was cooled on ice and the solids isolated following filtration. The solids were washed with ice-cold 5:1 (v/v) diethyl ether/*n*-hexane (30 mL) and pure **2a** isolated as an amorphous cream/white solid which was dried in vacuo.

Yield: 310 mg (77%) – from **1a**; 339 mg (84%) – from **1b**.

^{1}H NMR (400 MHz, DMSO-d_6) δ_H = 8.34 (1H, s, H8), 8.14 (1H, s, H2), 7.30 (2H, s, NH_2), 7.13 (2H, d, $^3J_{HH}$ = 8.5 Hz, ArH, *m*- to OMe), 6.81 (2H, d, $^3J_{HH}$ = 8.3 Hz, ArH, *o*- to OMe), 5.89 (1H, d, $^3J_{HH}$ = 5.3 Hz, H1'), 5.50 (1H, d, $^3J_{HH}$ = 5.8 Hz, 2'OH), 5.30 (1H, d, $^3J_{HH}$ = 5.0 Hz, 3'OH), 4.70-4.78 (1H, m, H2'), 4.15-4.20 (1H, m, H3'), 3.98-4.05 (1H, m, H4'), 3.71 (3H, s, OCH_3), 3.67 (2H, s, ArCH_2), 2.75-2.85 and 2.55-2.65 (2H, 2 x m, H5', H5'').

^{13}C NMR (101 MHz, DMSO-d_6) δ_C = 158.09, 156.07, 152.63, 149.40, 139.93, 130.11, 129.94 (2C), 119.19, 113.67 (2C), 87.61, 83.76, 72.62, 72.52, 54.98, 34.98, 33.15.

ES + MS ($C_{18}H_{21}N_5O_4S$), m/z = 404 (M + H); 426 (M + Na^+), calc. ($C_{18}H_{21}N_5O_4SNa$) 426.1212, found 426.1215.

m.p. (crystallised material) 183-186°C.

5′-*S*-(4-Methoxybenzyl)-5′-thiothymidine (**2b**) – MobSdT

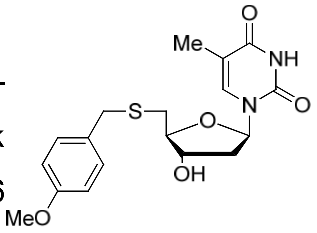

From TsT (**1c**). Under a gentle stream of argon, two zirconia-lined jars were each sequentially charged, in quick succession, with 5′-*O*-(*p*-toluenesulfonyl)thymidine (**1c**, 396 mg, 1.0 mmol), 4-methoxybenzylthiol (418 µL, 3.0 mmol, 3 equiv), 1,1,3,3-tetramethylguanidine (627 µL, 5.0 mmol, 5 equiv) and a 15 mm diameter zirconia ball. The vessels were sealed and vibrated at 30 Hz for 15 minutes and allowed to cool to ambient temperature. Each jar was extracted with aliquots (20 mL, 10 mL and 5 mL) of both water and ethyl acetate and the combined extractates diluted with ethyl acetate (600 mL) and 10% (w/v) KH_2PO_4 (aq) (300 mL).

The aqueous material was carefully acidified to pH 3 using 2 M HCl (aq) and the organics partitioned and subsequently washed with sat. $NaHCO_3$ (aq) (3 × 300 mL) and brine (300 mL). The ethyl acetate solution was then dried over anhydrous Na_2SO_4, filtered and reduced in vacuo. The crude solid residue was triturated with 5:1 (v/v) diethyl ether/*n*-hexane (150 mL) under sonication, the suspension was cooled on ice and the solids collected by filtration. The solids were then washed with ice-cold 5:1 (v/v) diethyl ether/*n*-hexane (150 mL) and dried in vacuo. Pure **2b** was isolated as an amorphous white solid.
Yield: 720 mg (95%).

From IdT (**1d**). Under a gentle stream of argon, a zirconia-lined jar was sequentially charged, in quick succession, with 5′-iodo-5′-deoxythymidine (**1d**, 131 mg, 0.30 mmol) and 4-methoxybenzylthiol (140 µL, 0.90 mmol, 3 equiv) and a 15 mm diameter zirconia ball. The vessel was vibrated at 30 Hz for 30 minutes and allowed to cool to ambient temperature. To the untransformed mixture, under argon, was added 1,1,3,3-tetramethylguanidine (209 µL, 1.5 mmol, 5 equiv) and vibration continued at 30 Hz for a further 30 minutes. The reaction mixture was extracted from the vessel into a separating funnel by interleaving washings (5 mL aliquots × 2) of water and ethyl acetate. The mixture was diluted with ethyl acetate (130 mL) and 10% (w/v) aqueous KH_2PO_4 (50 mL). The cloudy aqueous layer was carefully acidified to pH 3 using 2 M HCl (aq) during which procedure the solids dissolved. The organics were then partitioned, washed with brine (50 mL) and dried over anhydrous Na_2SO_4. The solution was filtered, reduced in vacuo and the solids trituated with 5:1 (v/v) diethyl ether/*n*-hexane (30 mL) under sonication. The suspension was cooled on ice, filtered and the solids washed with ice-cold 5:1 (v/v) diethyl ether/*n*-hexane (30 mL). The pure product (**2b**) - a white amorphous solid - was dried in vacuo.
Yield: 103 mg (90%).

From TsT (**1c**) – with expedited work-up. Under a gentle stream of argon, a zirconia-lined jar was charged, in quick succession, with 5′-*O*-(*p*-toluenesulfonyl)thymidine (**1c**, 396 mg, 1.0 mmol), 4-methoxybenzylthiol (418 µL, 3.0 mmol, 3 equiv), 1,1,3,3- tetramethylguanidine (627 µL, 5.0 mmol, 5 equiv) and a 15 mm diameter zirconia ball.

The vessel was sealed and vibrated at 30 Hz for 5 minutes and allowed to cool to ambient temperature. This reaction mixture jar was readily extracted from the jar in acetonitrile (20 mL) the extractate diluted with 10% (w/v) KH_2PO_4 (aq) (100 mL). The suspension was carefully acidified to pH 3 using 2 M HCl (aq) and sonicated to effect dispersion of the solids. The suspension was subsequently cooled on ice and the solids isolated by filtration. The solids were further washed with ice-cold water (50 mL), dried in vacuo over P_2O_5 and triturated with 5:1 (v/v) diethyl ether/*n*-hexane (30 mL) under sonication. The suspension was cooled on ice and the solids isolated following filtration. These solids were washed with ice-cold 5:1 diethyl ether/*n*-hexane (30 mL) and dried in vacuo to obtain pure **2b** as an amorphous white solid. Yield: 330 mg (87%).

^{1}H NMR (400 MHz, DMSO-d_6) δ_H = 11.30 (1H, s, N<u>H</u>), 7.47 (1H, s, H6), 7.22 (2H, d, $^3J_{HH}$ = 8.5 Hz, Ar<u>H</u>, *m*- to OMe), 6.87 (2H, d, $^3J_{HH}$ = 8.5 Hz, Ar<u>H</u>, *o*- to OMe), 6.17 (1H, ψt, $^3J_{HH}$ = 7.0 Hz, H1'), 5.34 (1H, d, $^3J_{HH}$ = 4.3 Hz, 3'O<u>H</u>), 4.09-4.22 (1H, m, H3'), 3.87-3.79 (1H, m, H4'), 3.73 (5H, s, OC<u>H</u>$_3$, ArC<u>H</u>$_2$), 2.65-2.78 and 2.60-2.65 (2H, 2 x m, H5', H5''), 2.15-2.30 and 1.95-2.10 (2H, 2 x m, H2', H2''), 1.78 (3H, s, C<u>H</u>$_3$).
^{13}C NMR (101 MHz, DMSO-d_6) δ_C = 163.66, 158.16, 150.46, 136.04, 130.12, 129.98 (2C), 113.76 (2C), 109.75, 85.23, 83.66, 72.41, 55.01, 37.95, 35.04, 33.10, 12.13.
ES + MS ($C_{18}H_{22}N_2O_5S$), m/z = 401 (M + Na$^+$), calc. ($C_{18}H_{22}N_2O_5SNa$) 401.1147, found 401.1122.

<u>5′-*S*-(4-Methoxybenzyl)-5′-thioguanosine (**2c**) – MobSdG</u>

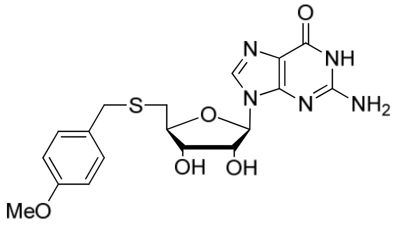

Under a gentle stream of argon, a zirconia-lined jar was sequentially charged, in quick succession, with 5′-iodo-5′-deoxyguanosine (**1e**, 393 mg, 1.0 mmol), 4-methoxybenzylthiol (418 µL, 3.0 mmol, 3 equiv), 1,1,3,3-tetramethylguanidine (627 µL, 5.0 mmol, 5 equiv) and a 15 mm diameter zirconia ball. The vessel was sealed and vibrated at 30 Hz for one hour and allowed to cool to ambient temperature. The reaction mixture was extracted from the vessel into a separating funnel by interleaving washings (25 mL and 10 mL aliquots) of water and ethyl acetate. The mixture was diluted with ethyl acetate (450 mL) and 10% (w/v) aqueous KH_2PO_4 (150 mL). The aqueous material was carefully acidified to pH 3 using 2M HCl (aq). During acidification, crystallisation of

a white solid was observed. The solid was isolated by filtration and washed successively with ethyl acetate (60 mL), water (50 mL) and a further aliquot of ethyl acetate (20 mL) to give pure **2c** as a white solid.
Yield: 380 mg (91%).

^{1}H NMR (400 MHz, DMSO-d_6) δ_H = 10.63 (1H, s, N1-H), 7.90 (1H, s, H8), 7.17 (2H, d, $^3J_{HH}$ = 8.6 Hz, ArH, *m*- to OMe), 6.84 (2H, d, $^3J_{HH}$ = 8.6 Hz, ArH, *o*- to O-CH_3), 6.46 (2H, br s, NH_2), 5.69 (1H, d, $^3J_{HH}$ = 5.9 Hz, H1'), 5.46 (1H, d, $^3J_{HH}$ = 5.9 Hz, 2'OH), 5.20 (1H, d, $^3J_{HH}$ = 5.1 Hz, 3'OH), 4.47-4.59 (1H, m, H2'), 4.01-4.11 (1H, m, H3'), 3.89-3.99 (1H, m, H4'), 3.72 (3H, s, OCH_3), 3.69 (2H, s, $ArCH_2$), 2.55-2.70 and 2.73-2.85 (2H, 2 x m, H5', H5'').
^{13}C NMR (101 MHz, DMSO-d_6) δ_C = 158.14, 156.78, 153.68, 151.42, 135.81, 130.15, 130.01 (2C), 116.78, 113.74 (2C), 86.60, 83.57, 72.65, 72.59, 55.02, 35.01, 33.24.
ES + MS ($C_{18}H_{21}N_5O_5S$), m/z = 442 (M + Na^+), calc. ($C_{18}H_{21}N_5O_5SNa$) 442.1161, found 442.1161.

5′-Deoxyadenosinyl-5′-selenocyanate (**3a**) - NCSedA

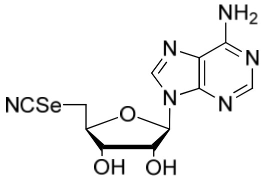

Under a gentle stream of argon, two zirconia-lined jars were each sequentially charged, in quick succession, with aliquots of 5′-*O*-(*p*-toluenesulfonyl)adenosine (**1b**, 126 mg, 0.30 mmol or 253 mg, 0.60 mmol), potassium selenocyanate (130 mg, 0.90 mmol or 260 mg, 1.8 mmol; 3 equiv), anhydrous DMF (50 µL or 100 µL) and a 15 mm diameter zirconia ball. The vessels were vibrated at 30 Hz for eleven hours and allowed to cool to ambient temperature. The reaction mixtures were extracted from the vessel in methanol (30 and 50 mL aliquots respectively). Both reaction mixtures were combined and stirred at room temperature for 30 minutes under argon in the presence of benzyl bromide (236 µL, 1.98 mmol, 2.2 equiv). The quenched reaction mixture was adsorbed onto silica gel and subject to column chromatography eluting with 5–15% (v/v) methanol in DCM. Fractions containing **3a** (contaminated with a *p*-toluene sulphonate salt) were combined and reduced in vacuo. The tosylate-contaminated solids (104 mg) were partitioned between EtOAc (30 mL) and deionised H_2O (15 mL) followed by brine (10 mL). The combined aqueous phases

were back-extracted with EtOAc (10 × 10 mL) and the organics combined, dried over Na_2SO_4 and filtered. The solvent was removed in vacuo to give pure product **3a** as a white amorphous solid.
Yield = 65 mg (20%).

^{1}H NMR (400 MHz, DMSO-d_6) δ_H = 8.36 (1H, s, H8), 8.15 (1H, s, H2), 7.29 (2H, s, NH_2), 5.93 (1H, d, $^{3}J_{HH}$ = 5.9 Hz, H1'), 5.58 (1H, d, $^{3}J_{HH}$ = 6.1 Hz, 2'O<u>H</u>), 5.49 (1H, d, $^{3}J_{HH}$ = 4.9 Hz, 3'O<u>H</u>), 4.75-4.86 (1H, m, H2'), 4.22-4.29 (1H, m, H3'), 4.11-4.20 (1H, m, H4'), 3.47-3.55 and 3.31-3.40 (2H, 2 x m, H5', H5'').
^{13}C NMR (151 MHz, DMSO-d_6) δ_C = 156.09, 152.60, 149.33, 140.09, 119.28, 104.55, 87.70, 83.42, 72.73, 40.05, 31.62.
^{77}Se NMR (114 MHz, DMSO-d_6, external D_2O lock) δ_{Se} = 188.00 – 188.53 (m).
ES+ MS ($C_{11}H_{12}N_6O_3{}^{80}Se$), m/z = 356 (M + H^+), calc. ($C_{11}H_{13}N_6O_3{}^{80}Se$) 356.0215, found 329.0197.

<u>5′-Deoxythymidinyl-5′-selenocyanate (**3b**) – NCSedT</u>

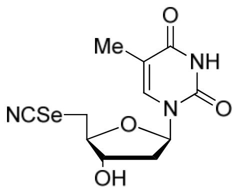

<u>From TsT (**1c**).</u> Under a gentle stream of argon, two zirconia-lined jars were each sequentially charged, in quick succession, with 5′-*O*-tosylthymidine (**1c**, 237 mg, 0.60 mmol), potassium selenocyanate (259 mg, 1.8 mmol, 3 equiv), anhydrous DMF (100 µL) and a 15 mm diameter zirconia ball. The vessels were vibrated at 30 Hz for nine hours and allowed to cool to ambient temperature. The reaction mixtures were extracted from the vessels in methanol (50 mL aliquots) and the combined extractates were stirred under ambient conditions during addition of benzylbromide (310 µL, 2.6 mmol, 2.2 equiv). These conditions were maintained for 30 minutes. The reaction mixture was reduced in vacuo and adsorbed onto silica gel prior to purification by column chromatography eluting with 5–12.5% (v/v) methanol in DCM. Fractions containing pure **3b** were combined and reduced in vacuo to give pure product as a pale brown amorphous solid.
Yield = 277 mg (70%).

From IdT (**1d**). Under a gentle stream of argon, two zirconia-lined jars were each sequentially charged, in quick succession, with 5′-iodo-5′-deoxythymidine (**1d**, 211 mg, 0.60 mmol), potassium selenocyanate (259 mg, 1.8 mmol, 3 equiv), anhydrous DMF (100 μL) and a 15 mm diameter zirconia ball. The vessels were vibrated at 30 Hz for nine hours and allowed to cool to ambient temperature. The reaction mixtures were extracted from the vessels in methanol (50 mL aliquots) and the combined extractates were stirred under ambient conditions during addition of benzylbromide (310 μL, 2.6 mmol, 2.2 equiv). These conditions were maintained for 30 minutes. The reaction mixture was reduced in vacuo and adsorbed onto silica gel prior to purification by column chromatography eluting with 5–15% (v/v) methanol in DCM. Fractions containing pure **3b** were combined and reduced in vacuo to give pure product as a pale brown, electrostatically-charged amorphous solid.
Yield = 350 mg (88%).

^{1}H NMR (400 MHz, DMSO-d_6) δ_H = 11.32 (1H, s, N$\underline{H}$), 7.50 (1H, s, H6), 6.20 (1H, dd, $^{3}J_{HH}$ = 8.2, 6.1 Hz, H1′), 5.49 (1H, brs, 3′O$\underline{H}$), 4.26 (1H, m, H4′), 3.98 (1H, m, H3′), 3.36-3.46 and 3.21-3.28 (2H, 2 x m, H5′, H5″), 2.19-2.30 and 2.05-2.15 (2H, 2 x m, H2′, H2″), 1.79 (3H, s, C$\underline{H}_3$).
^{13}C NMR (101 MHz DMSO-d_6) δ_C = 163.67, 150.41, 136.23, 109.72, 104.69, 85.17, 84.15, 72.55, 38.06, 31.50, 12.02.
^{77}Se NMR (114 MHz, DMSO-d_6, external D_2O lock) δ_{Se} = 187.40 – 187.90 (m).
ES+ MS ($C_{11}H_{13}N_3O_4{}^{80}Se$), m/z = 332 (M + H$^+$), 354 (M + Na$^+$), calc. ($C_{11}H_{12}N_3O_4{}^{80}SeNa$) 353.9966, found 329.9969.

5′-Deoxyguanosinyl-5′-selenocyanate (**3c**) - NCSedG

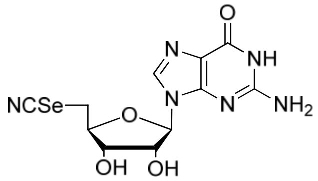

Under a gentle stream of argon, a zirconia-lined jar was sequentially charged, in quick succession, with 5′-iodo-5′-deoxyguanosine (**1e**, 118 mg, 0.30 mmol), potassium selenocyanate (130 mg, 0.90 mmol, 3 equiv) and a 15 mm diameter zirconia ball. The vessel was vibrated at 30 Hz for one hour and allowed to cool to ambient temperature. The crude reaction mixture was extracted from the vessel either as a solid for analysis by mass spectrometry or in DMSO-d_6 (0.6 mL) under argon for NMR investigations.

^{1}H NMR (400 MHz, DMSO-d_6) δ_H = 7.90 (1H, s, H8), 6.46 (2H, br s, N<u>H</u>$_2$), 5.72 (1H, d, $^{3}J_{HH}$ = 4 Hz, H1'), 4.61-4.64 (1H, t, $^{3}J_{HH}$ = 8 Hz H2'), 4.15-4.17 (1H, m, H3'), 4.08-4.12 (1H, m, H4'), 3.29-3.34 and 3.49-3.51 (2H, 2 x m, H5', H5'').
^{77}Se NMR (114 MHz, DMSO-d_6, external D_2O lock) δ_{Se} = 282.5 – 283.5 (m, [<u>Sed</u>G]$_2$), 187.12 – 186.34 (m, NC<u>Sed</u>G), -275.60 (K<u>SeC</u>N in DMSO-d_6)
ES- MS ($C_{11}H_{12}N_6O_4{}^{80}Se$), m/z = 371 (M - H^+), 743 (2M - H^+), calc. ($C_{11}H_{11}N_6O_4{}^{80}Se$) 371.0007, found 371.0007 calc. ($C_{22}H_{23}N_{12}O_8{}^{80}Se_2$) 743.0097, found 743.0081.

<u>Benzyl selenocyanate - BnSeCN</u>

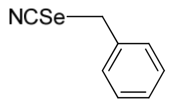

Under a gentle stream of argon, a zirconia-lined jar was sequentially charged, in quick succession, with benzyl bromide (1.19 mL, 1.71 g, 10 mmol), potassium selenocyanate (1.585 g, 11 mmol, 1.1 equiv) and a 15 mm diameter zirconia ball. The vessel was vibrated at 30 Hz for one hour and allowed to cool to ambient temperature. The reaction mixture was extracted from the vessel into a separating funnel by interleaving washings of ethyl acetate (40 mL and 2 × 15 mL) and water (2 × 15 mL). The extractate was partitioned between ethyl acetate (100 mL) and water (100 mL) and the organics further washed with satd. aqueous sodium hydrogen carbonate (2 × 100 mL) and brine (100 mL).
The organics were dried over Na_2SO_4, filtered and reduced in vacuo to give BnSeCN as a pale yellow solid.
Yield: 1.90 g, (97%).

^{1}H NMR (400 MHz, DMSO-d_6) δ_H = 7.35-7.42 (4H, m, 4 x Ar<u>H</u>), 7.28-7.34 (1H, m, Ar<u>H</u>), 4.32 (1H, $^{2}J_{HSe}$ = 18.4 Hz).
^{13}C NMR (101 MHz, DMSO-d_6) δ_C = 138.28, 128.83 (2C), 128.56 (2C), 127.78, 104.82, 32.59 ($^{1}J_{CSe}$ = 53.2 Hz).
^{77}Se NMR (114 MHz, DMSO-d_6, external D_2O lock) δ_{Se} = 306.20 (t, $^{2}J_{HSe}$ = 16.0 Hz).

<u>4-Methoxybenzyl selenocyanate - MobSeCN</u>

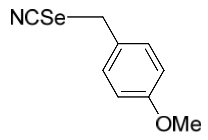

Under a gentle stream of argon, a zirconia-lined jar was sequentially charged, in quick succession, with 4-methoxybenzyl chloride (1.36

mL, 1.57 g, 10 mmol) and potassium selenocyanate (1.585 g, 11 mmol, 1.1 equiv) and a 15 mm diameter zirconia ball. The vessel was vibrated at 30 Hz for one hour and allowed to cool to ambient temperature. The reaction mixture was extracted from the vessel into a separating funnel by interleaving washings of ethyl acetate (40 mL and 2 × 15 mL) and water (2 × 15 mL). The organic solution was diluted with ethyl acetate (330 mL) and washed with water (170 mL), satd. aqueous sodium hydrogen carbonate (2 × 200 mL) and brine (200 mL).

The organics were dried over Na_2SO_4, filtered and solvents evaporated in vacuo to give MobSeCN as a pale pink amorphous solid.

Yield: 2.23 g (99%).

^{1}H NMR (400 MHz, DMSO-d_6) δ_H = 7.27-7.34 (2H, m, ArH), 6.90-6.95 (2H, m, ArH), 4.30 (2H, $^2J_{HSe}$ = 18.6 Hz), 3.75 (3H, s, CH_3).

^{13}C NMR (101 MHz, DMSO-d_6) δ_C = 158.92, 130.16 (2C), 130.00, 113.99 (2C), 104.93, 55.09, 32.70 ($^1J_{CSe}$ = 52.6 Hz).

^{77}Se NMR (114 MHz, DMSO-d_6, external D_2O lock) δ_{Se} = 321.43 (t, $^2J_{HSe}$ = 18.2 Hz).

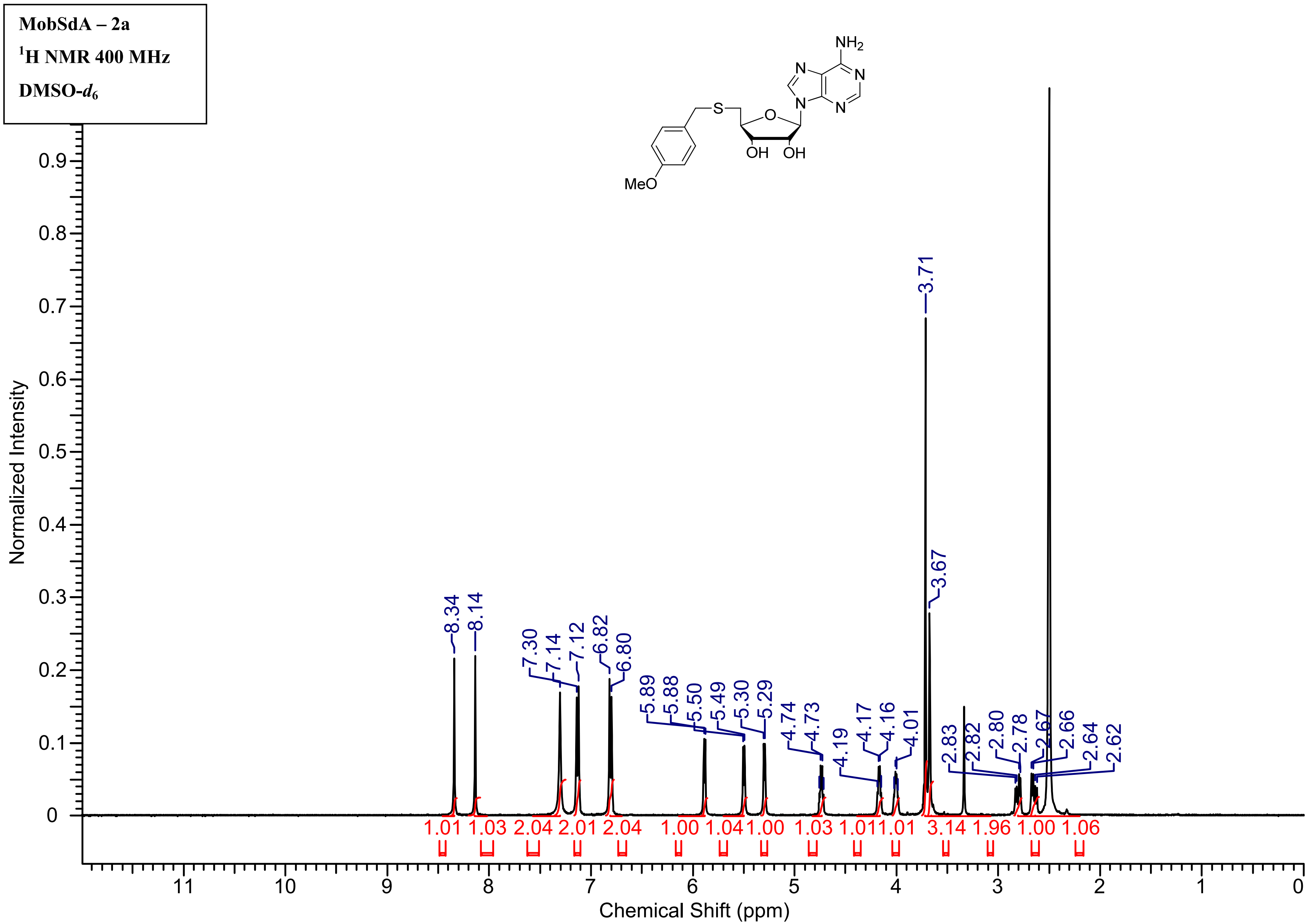
MobSdA – 2a
1H NMR 400 MHz
DMSO-d6
NH2
N
N
N
N
O
S
OH OH
MeO
8.34
8.14
7.30
7.14
7.12
6.82
6.80
5.89
5.88
5.50
5.49
5.30
5.29
4.74
4.73
4.19
4.17
4.16
4.01
3.71
3.67
2.83
2.82
2.80
2.78
2.67
2.66
2.64
2.62
1.01 1.03 2.04 2.01 2.04 1.00 1.04 1.00 1.03 1.01 1.01 3.14 1.96 1.00 1.06
Normalized Intensity
Chemical Shift (ppm)

MobSdA – 2a
^{1}H NMR 400 MHz + D_2O
DMSO-d_6

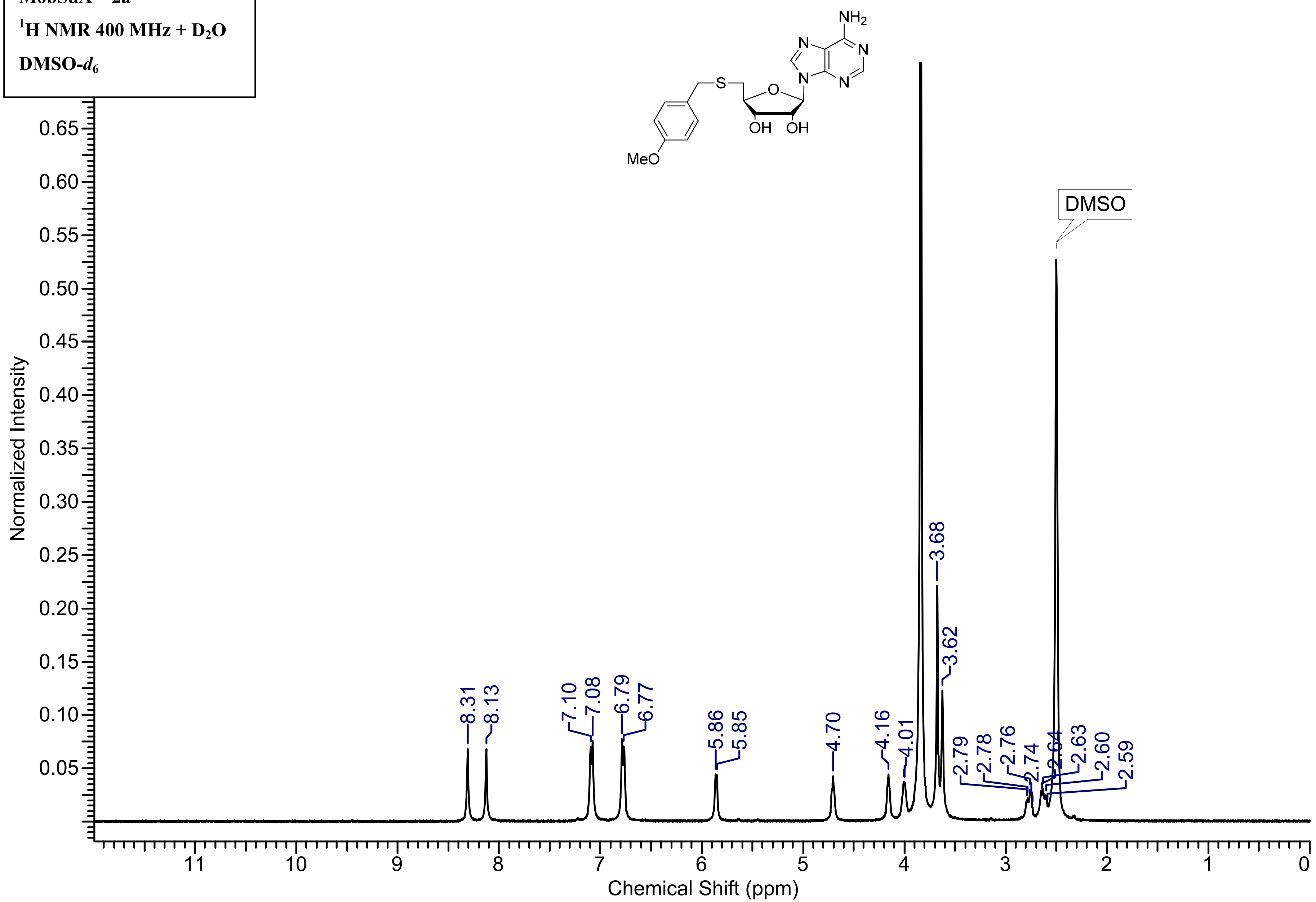

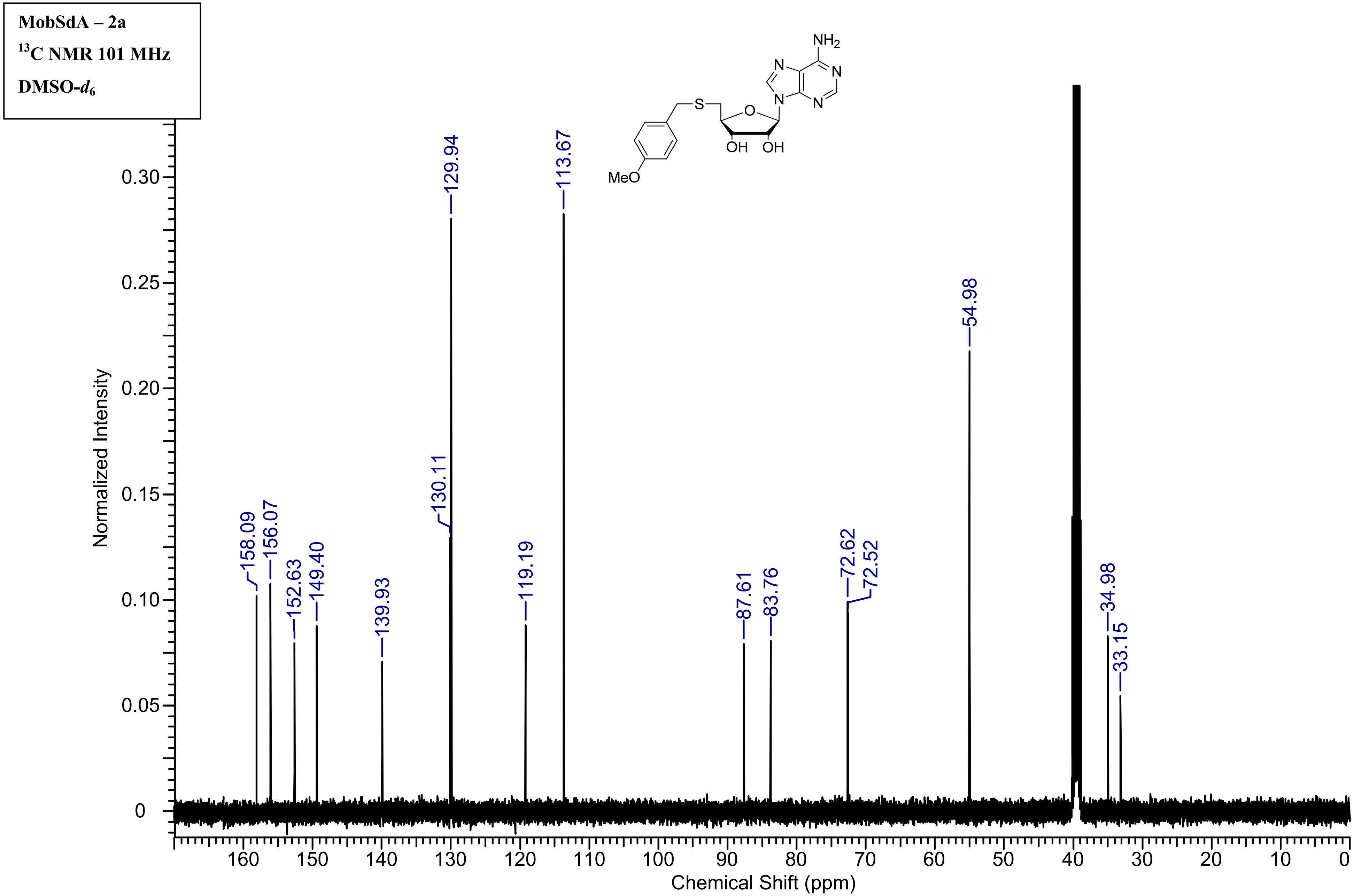
MobSdA – 2a
^{13}C NMR 101 MHz
DMSO-d_6
NH2
N
N
N
N
S
O
OH
OH
MeO
158.09
156.07
152.63
149.40
139.93
130.11
129.94
119.19
113.67
87.61
83.76
72.62
72.52
54.98
34.98
33.15
Normalized Intensity
Chemical Shift (ppm)
0.30
0.25
0.20
0.15
0.10
0.05
0
160
150
140
130
120
110
100
90
80
70
60
50
40
30
20
10
0

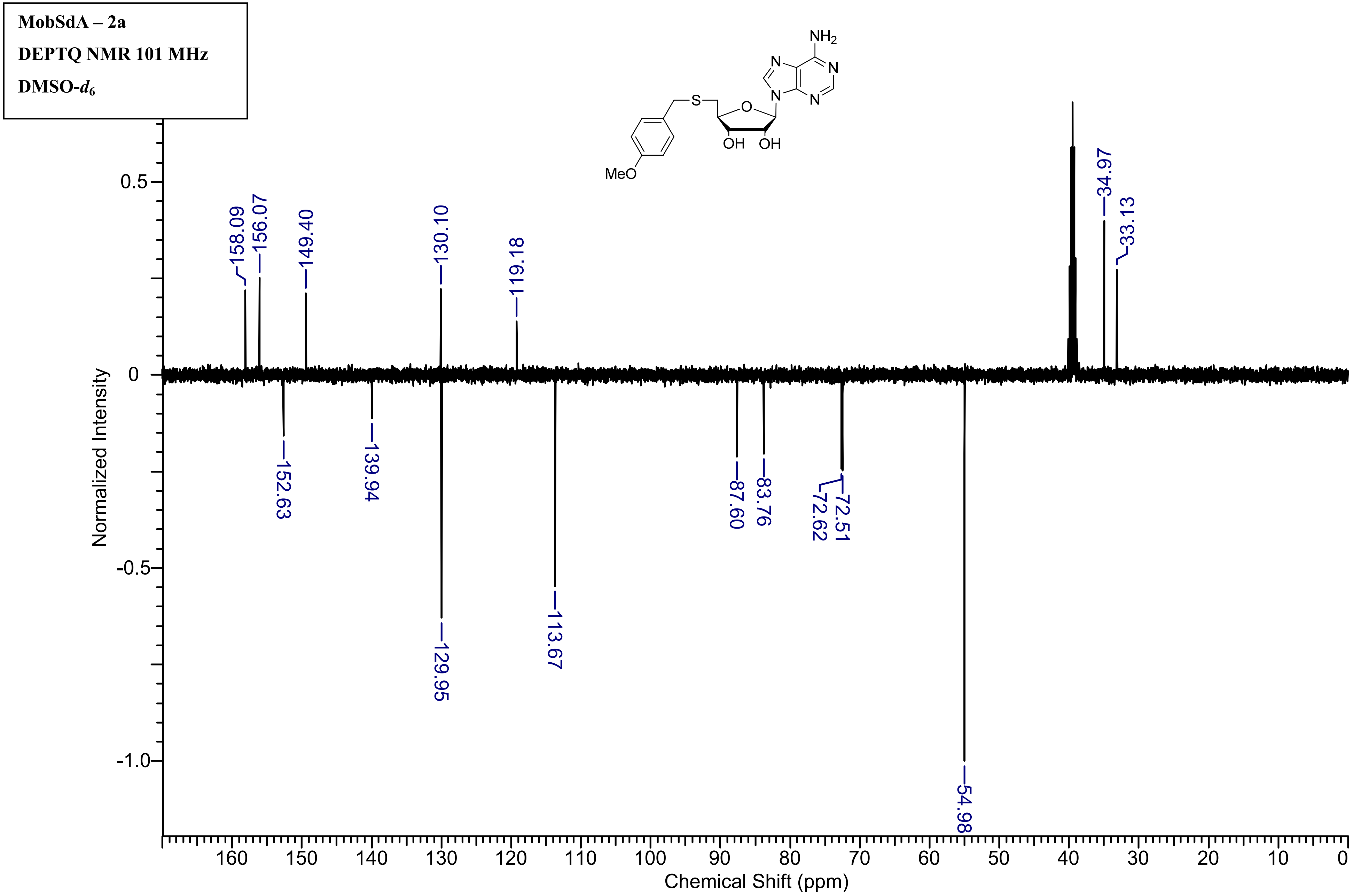
MobSdA – 2a
DEPTQ NMR 101 MHz
DMSO-d_6
NH_2
N
N
N
N
S
O
OH
OH
MeO
158.09
156.07
149.40
130.10
119.18
34.97
33.13
152.63
139.94
129.95
113.67
87.60
83.76
72.62
72.51
54.98
0.5
0
-0.5
-1.0
Normalized Intensity
160
150
140
130
120
110
100
90
80
70
60
50
40
30
20
10
0
Chemical Shift (ppm)

MobSdA – 2a

HRMS (ESI, positive ion)

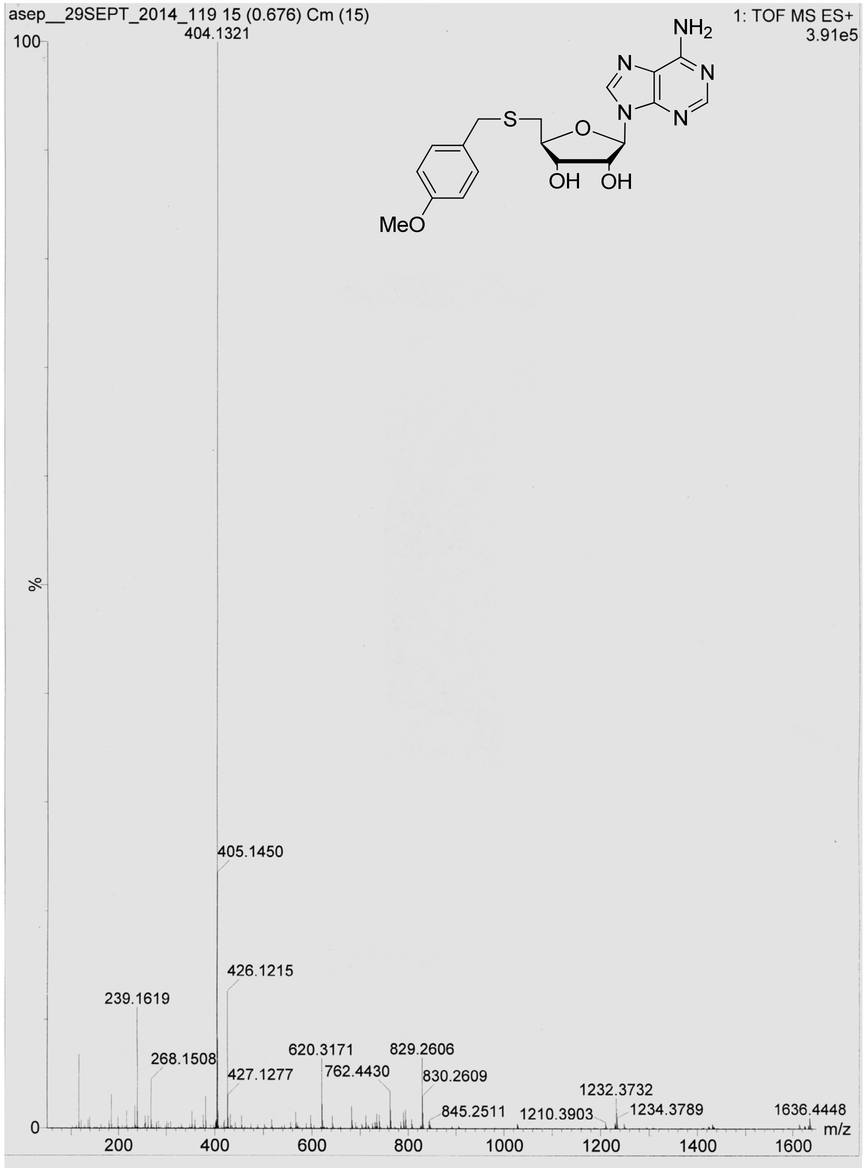

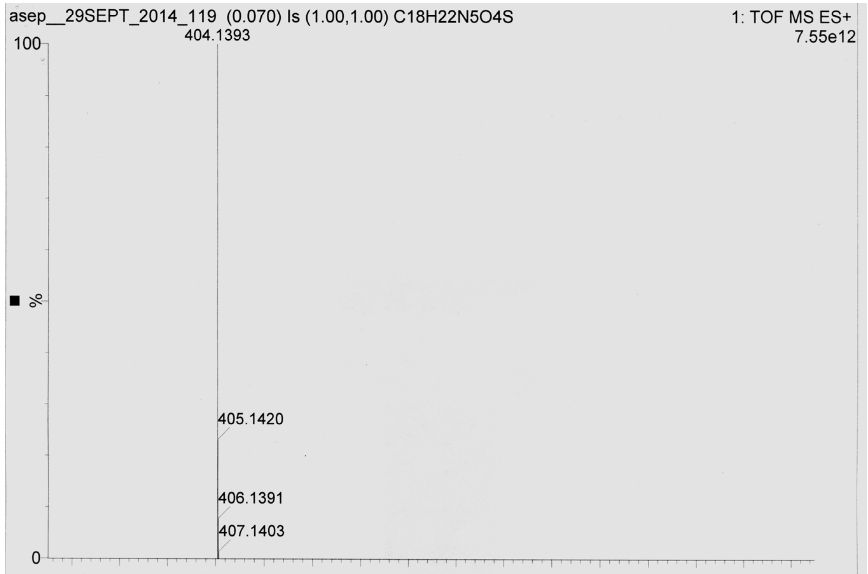

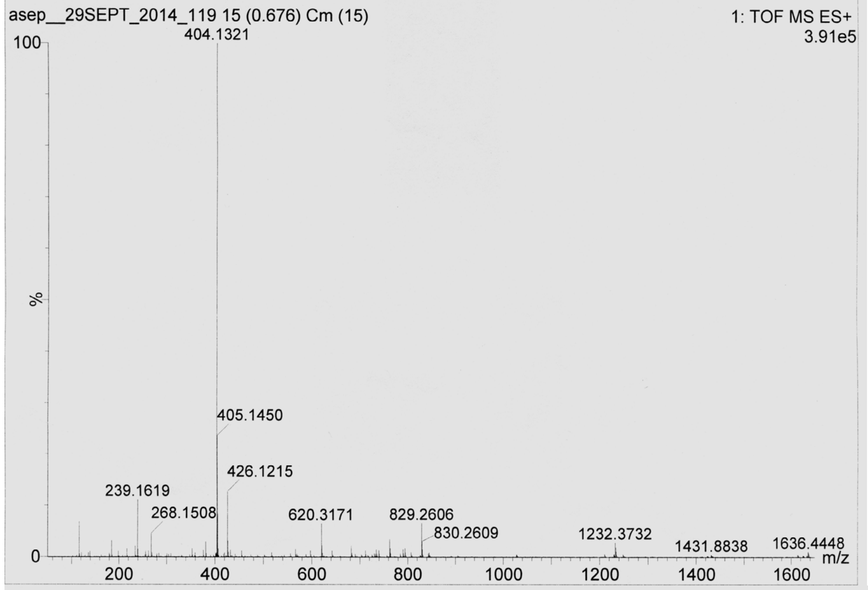

MobSdT – 2b
^{1}H NMR 400 MHz
DMSO-d_6

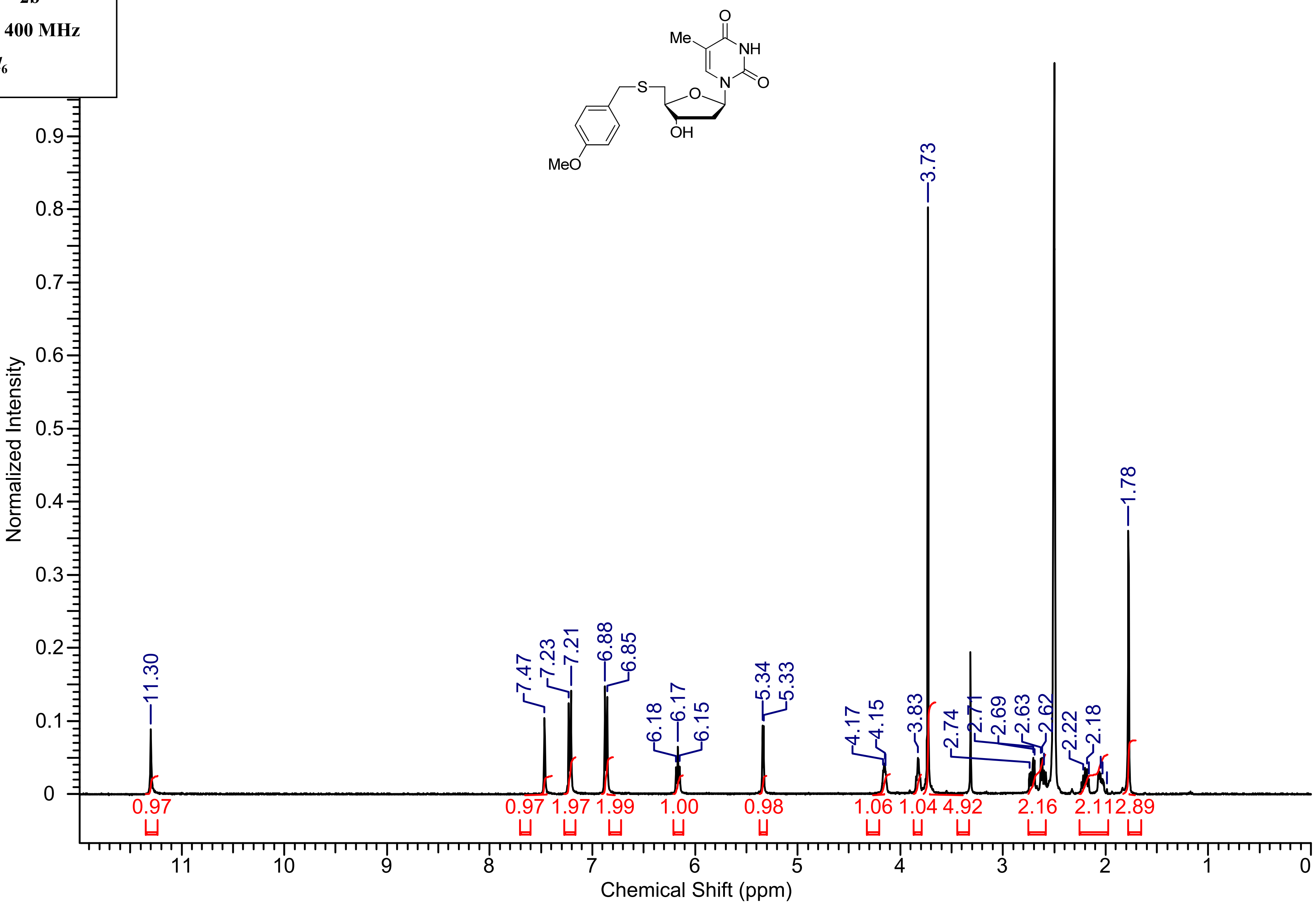

MobSdT – 2b

^{1}H NMR 400 MHz + D_2O

DMSO-d_6

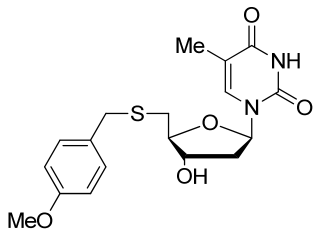

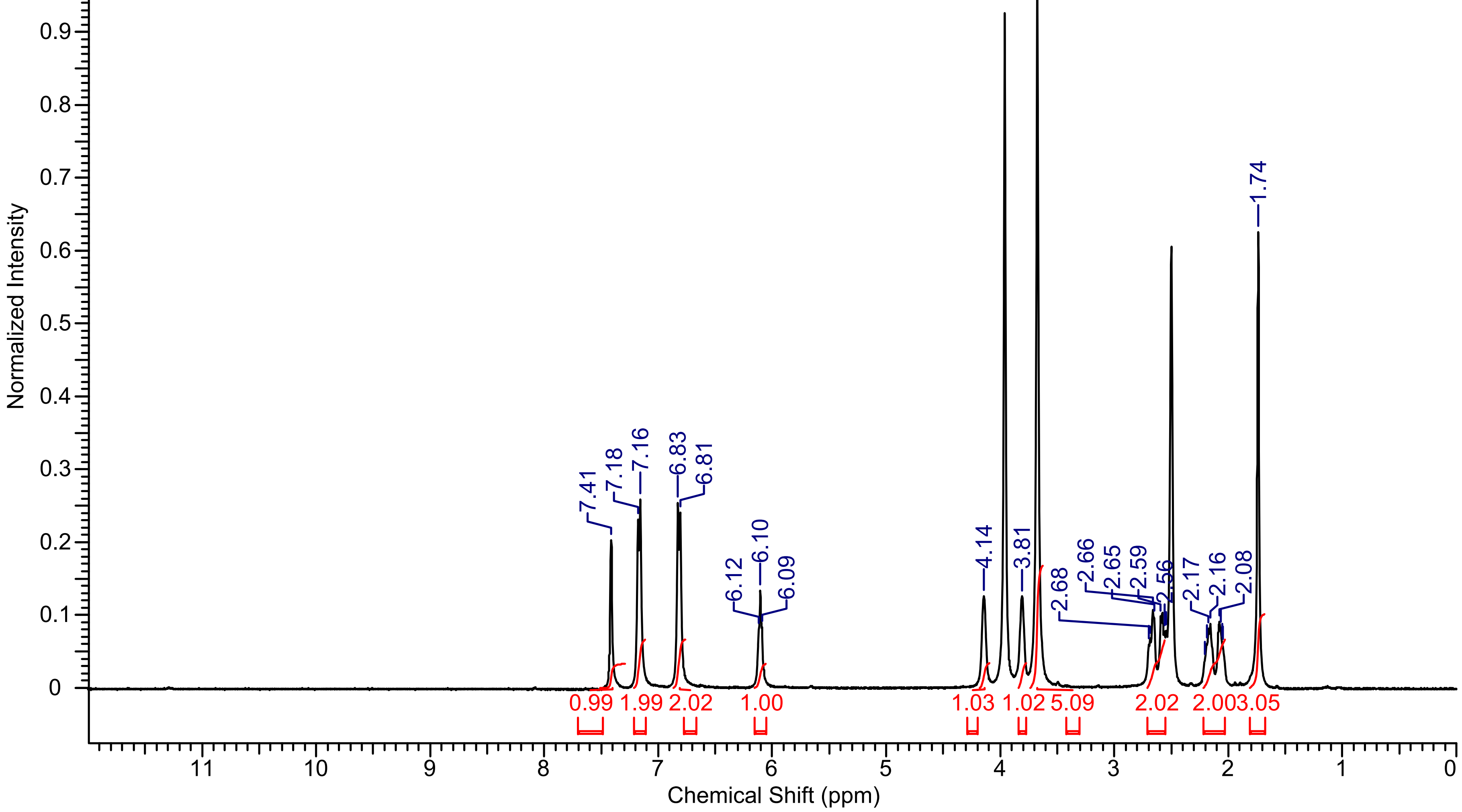

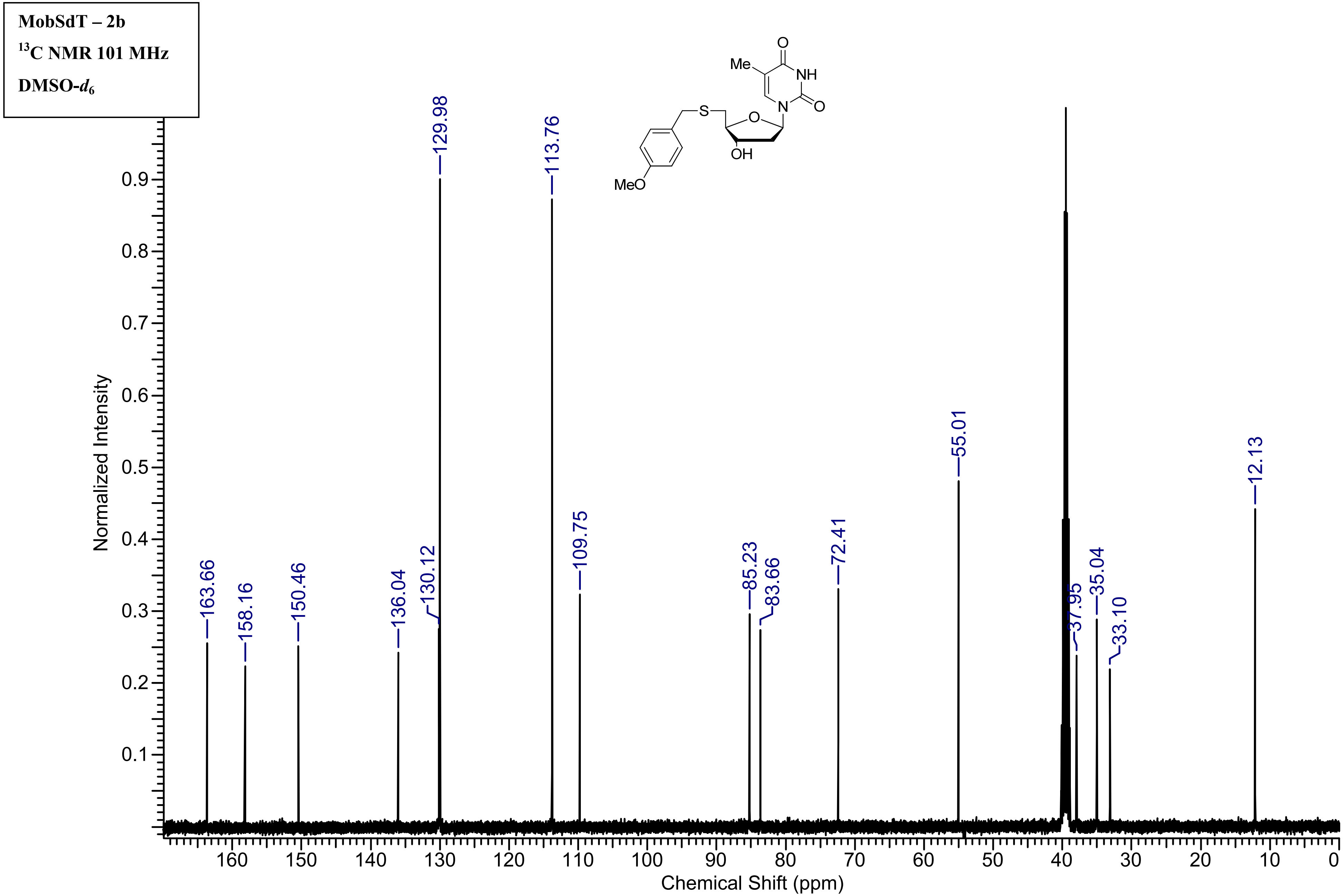

MobSdT – 2b
13C NMR 101 MHz
DMSO-d6
Me
O
NH
N
O
S
O
OH
MeO
129.98
113.76
163.66
158.16
150.46
136.04
130.12
109.75
85.23
83.66
72.41
55.01
37.95
35.04
33.10
12.13
Normalized Intensity
Chemical Shift (ppm)

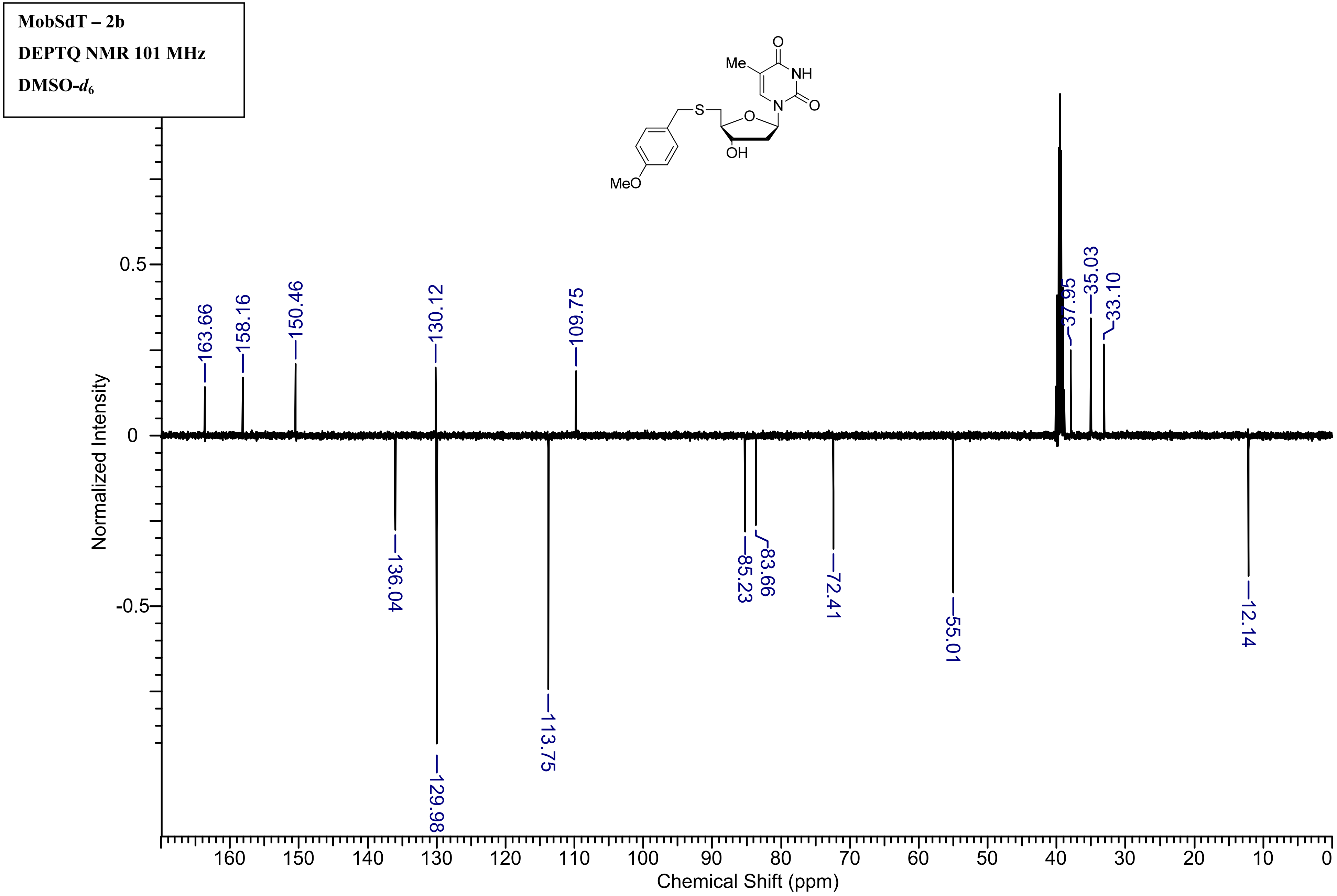
MobSdT – 2b
DEPTQ NMR 101 MHz
DMSO-d_6
Me
O
NH
N
O
S
O
OH
MeO
163.66
158.16
150.46
130.12
109.75
37.95
35.03
33.10
136.04
129.98
113.75
85.23
83.66
72.41
55.01
12.14
0.5
0
-0.5
Normalized Intensity
160
150
140
130
120
110
100
90
80
70
60
50
40
30
20
10
0
Chemical Shift (ppm)

MobSdT – 2b

HRMS (ESI, positive ion)

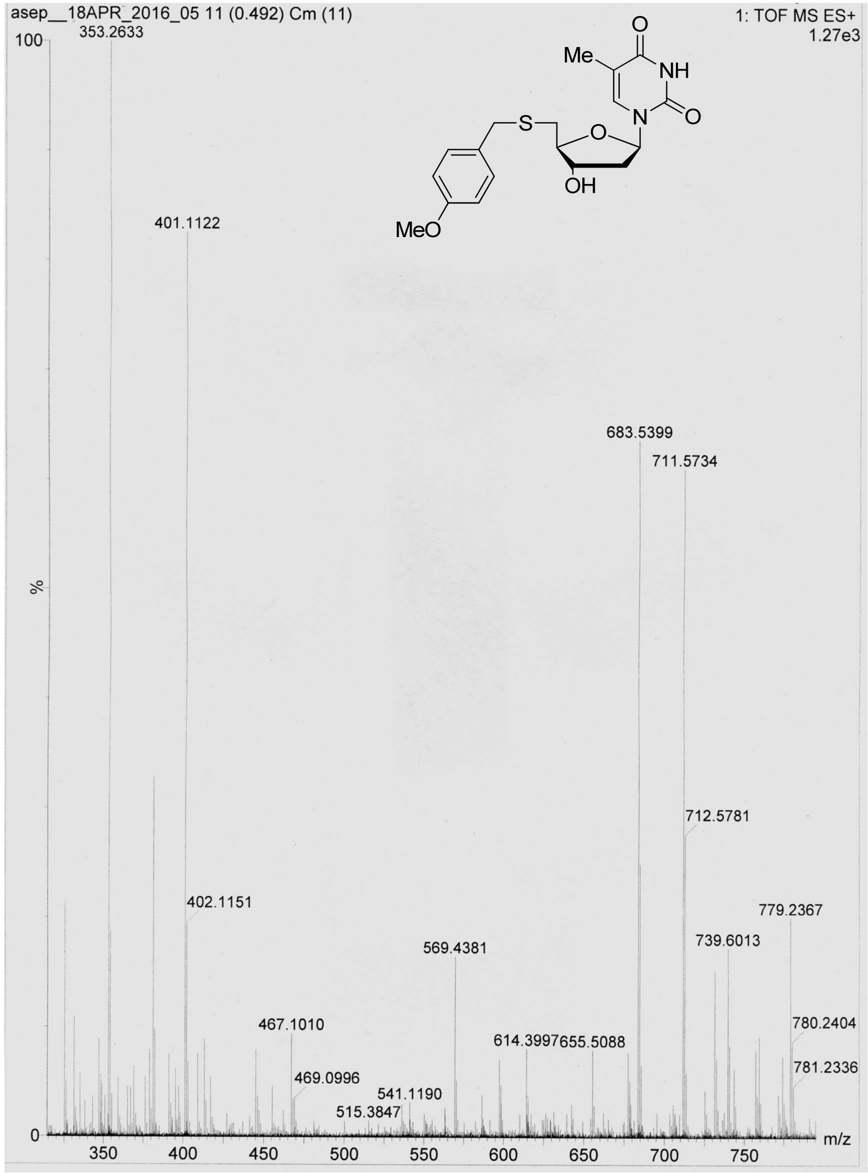

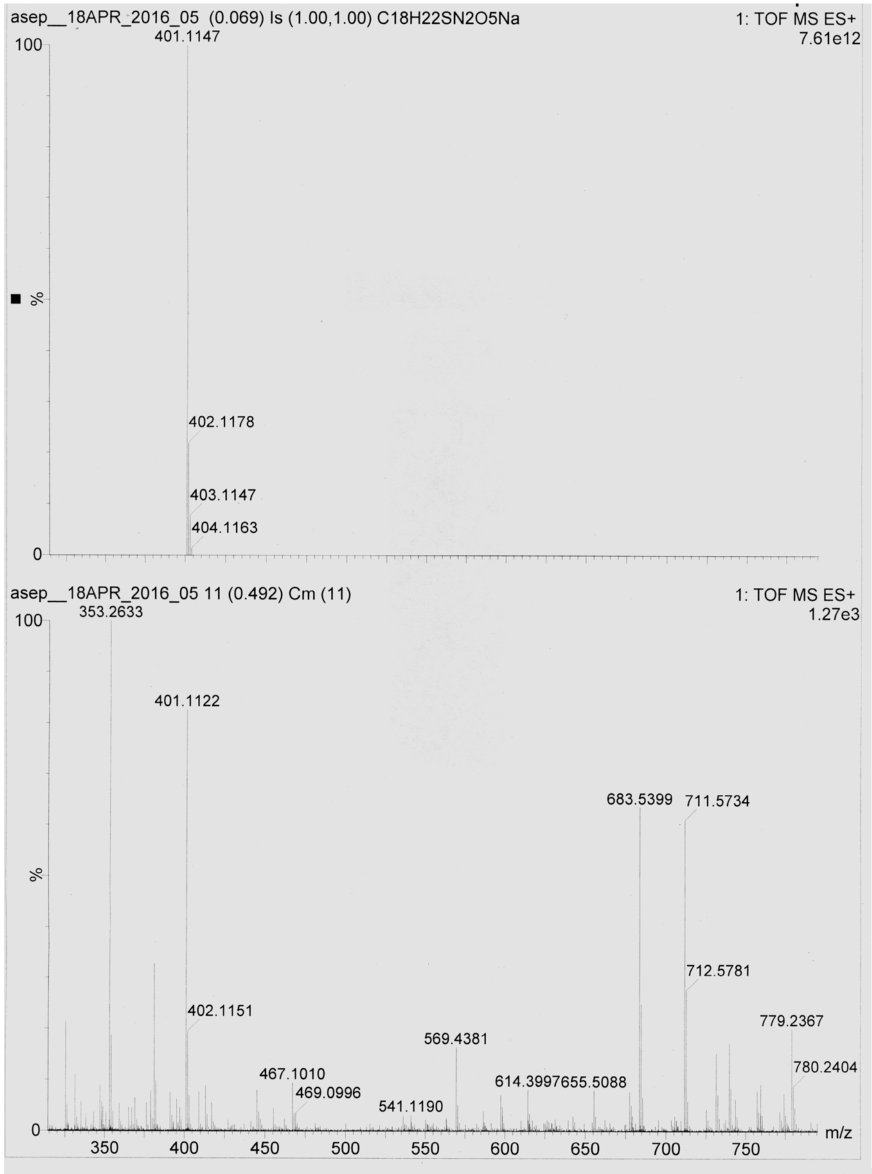

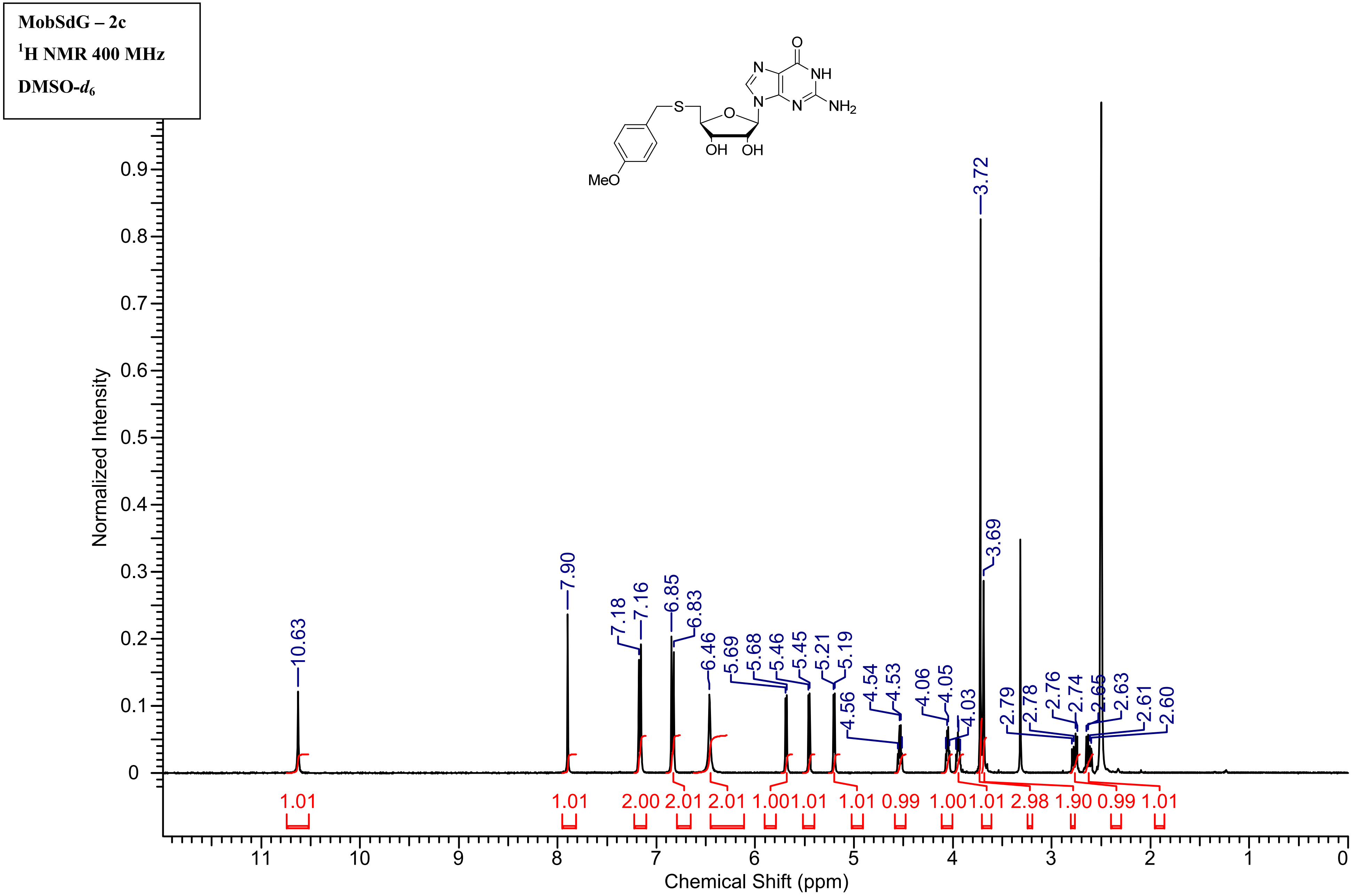

MobSdG – 2c
1H NMR 400 MHz
DMSO-d6
MeO
S
O
OH
OH
N
N
NH
N
NH2
O
Normalized Intensity
Chemical Shift (ppm)
10.63
7.90
7.18
7.16
6.85
6.83
6.46
5.69
5.68
5.46
5.45
5.21
5.19
4.56
4.54
4.53
4.06
4.05
4.03
3.72
3.69
2.79
2.78
2.76
2.74
2.65
2.63
2.61
2.60
1.01
1.01
2.00
2.01
2.01
1.00
1.01
1.01
0.99
1.00
1.01
2.98
1.90
0.99
1.01

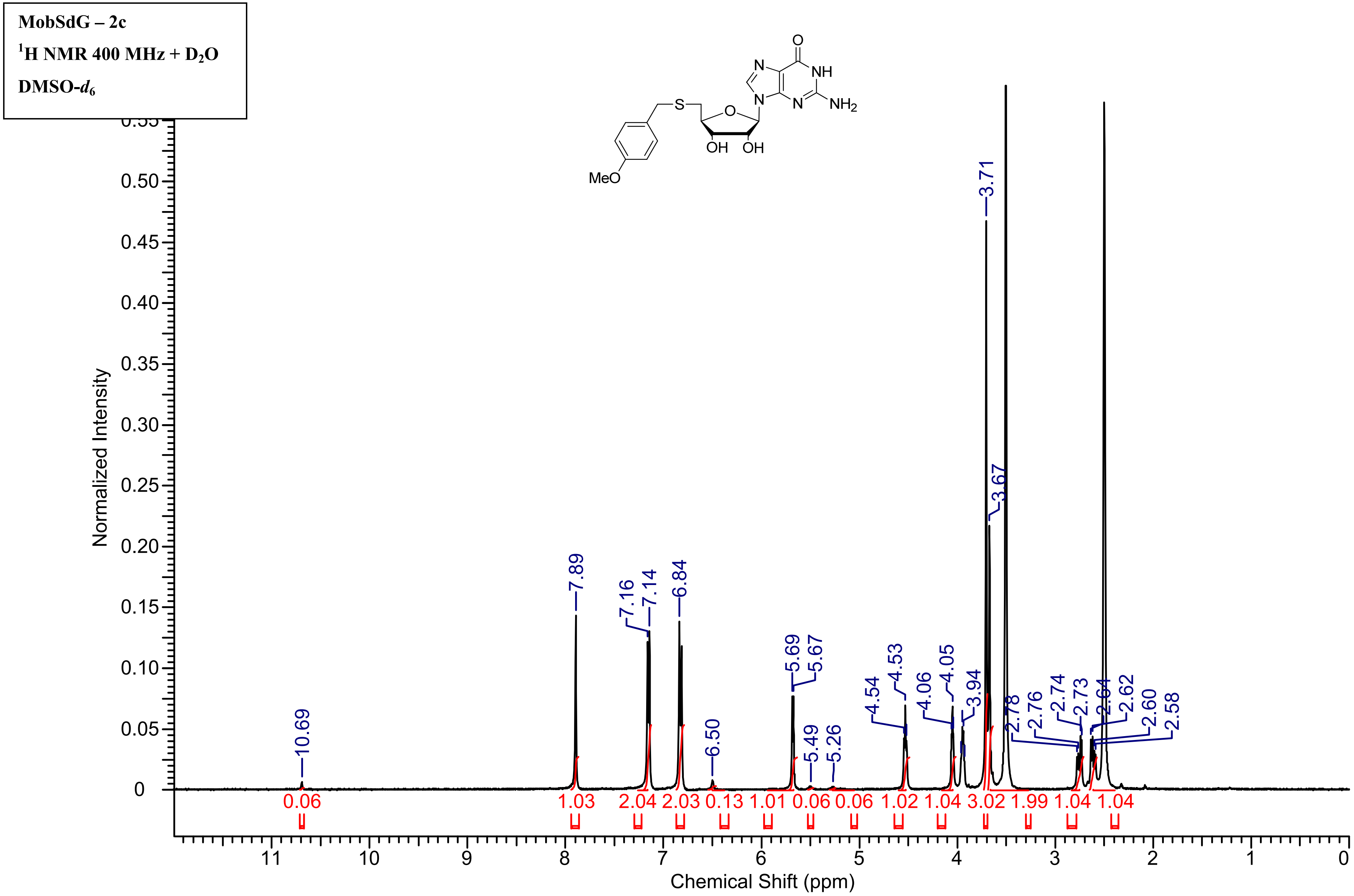
MobSdG – 2c
¹H NMR 400 MHz + D₂O
DMSO-d₆
O
N
NH
N
N
NH₂
S
O
OH OH
MeO
Normalized Intensity
0.50
0.45
0.40
0.35
0.30
0.25
0.20
0.15
0.10
0.05
0
10.69
7.89
7.16
7.14
6.84
6.50
5.69
5.67
5.49
5.26
4.54
4.53
4.06
4.05
3.94
3.71
3.67
2.78
2.76
2.74
2.73
2.64
2.62
2.60
2.58
0.06
1.03
2.04
2.03
0.13
1.01
0.06
0.06
1.02
1.04
3.02
1.99
1.04
1.04
11
10
9
8
7
6
5
4
3
2
1
0
Chemical Shift (ppm)

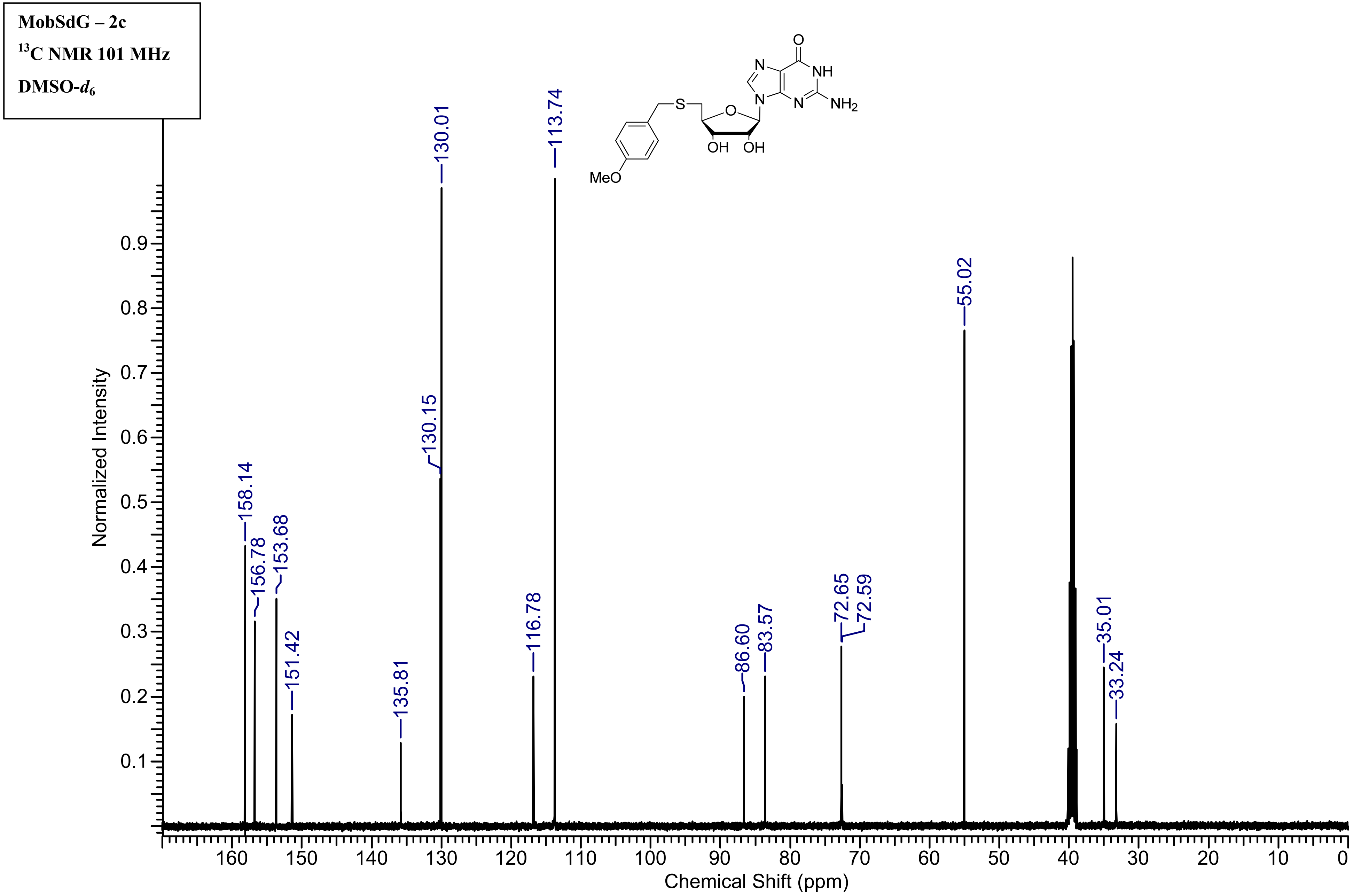
MobSdG – 2c
^{13}C NMR 101 MHz
DMSO-d_6
MeO
S
O
N
NH
NH2
OH OH
158.14
156.78
153.68
151.42
135.81
130.15
130.01
116.78
113.74
86.60
83.57
72.65
72.59
55.02
35.01
33.24
Normalized Intensity
Chemical Shift (ppm)

MobSdG – 2c

DEPTQ NMR 101 MHz

DMSO-d_6

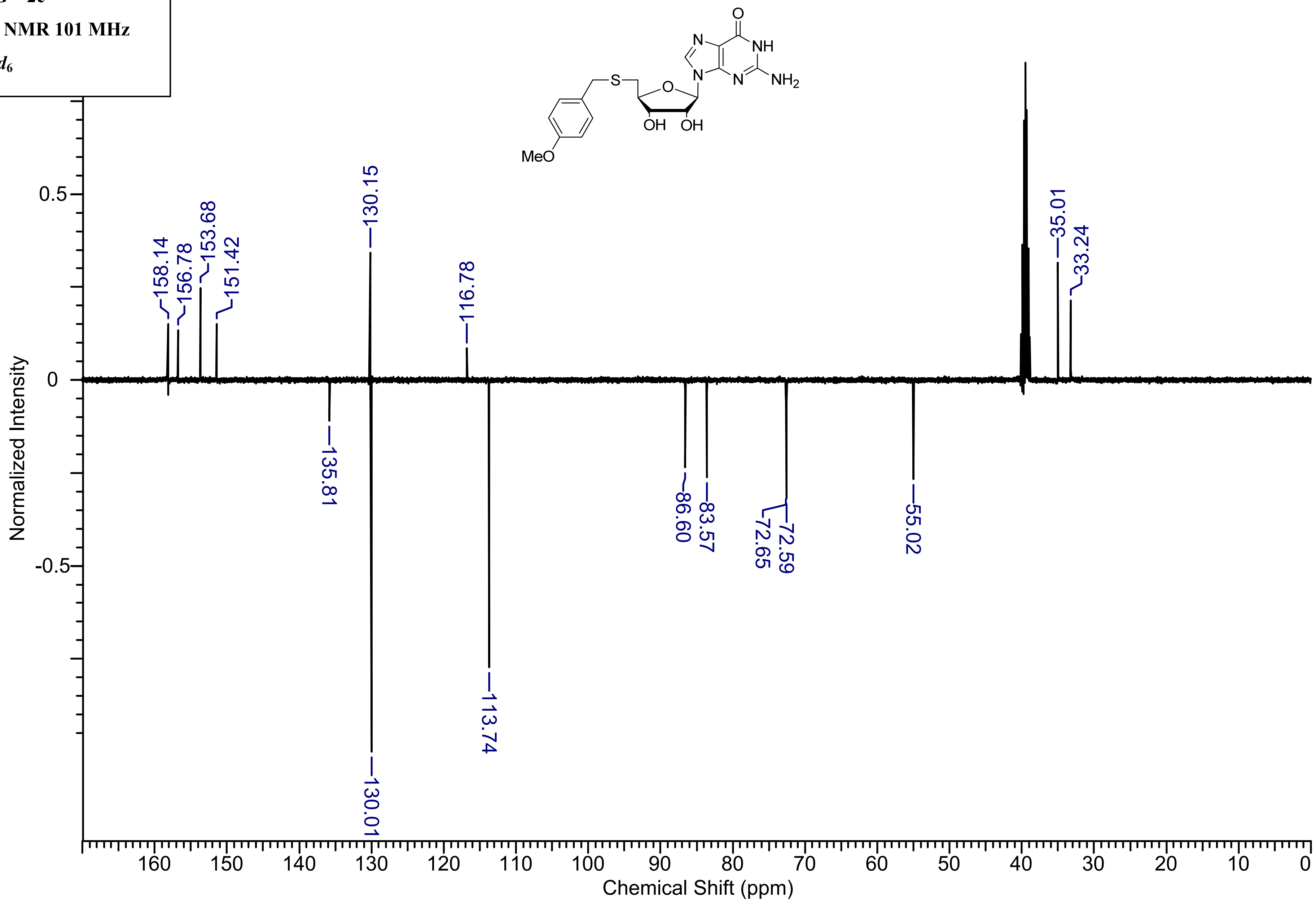

MobSdG – 2c

HRMS (ESI, positive ion)

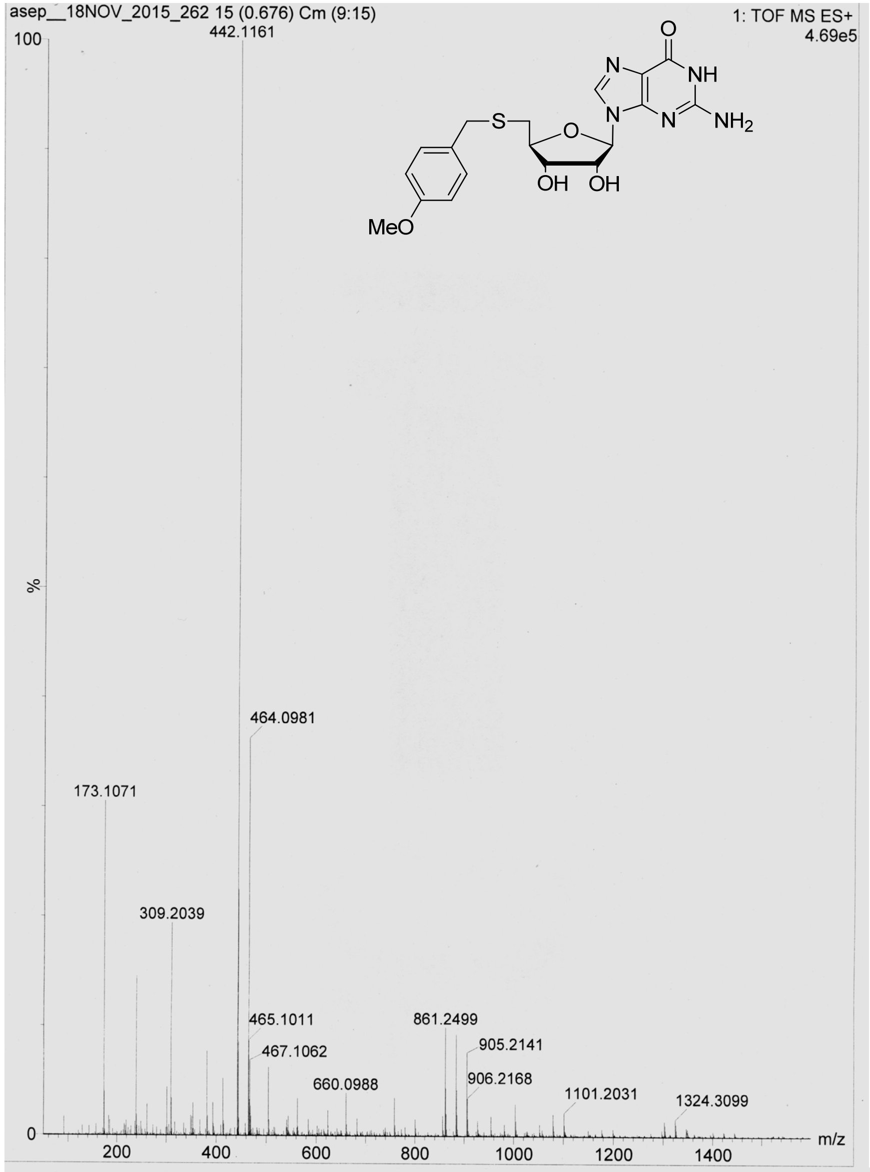

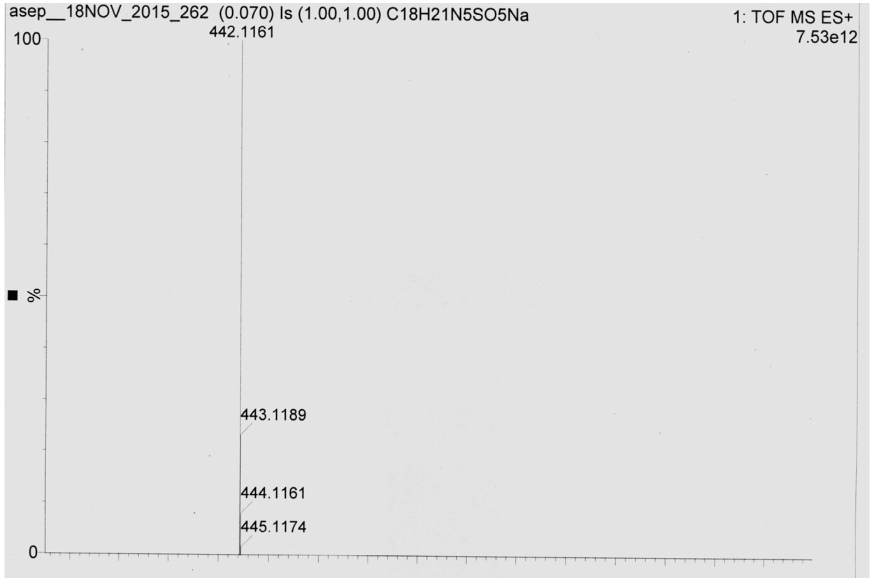

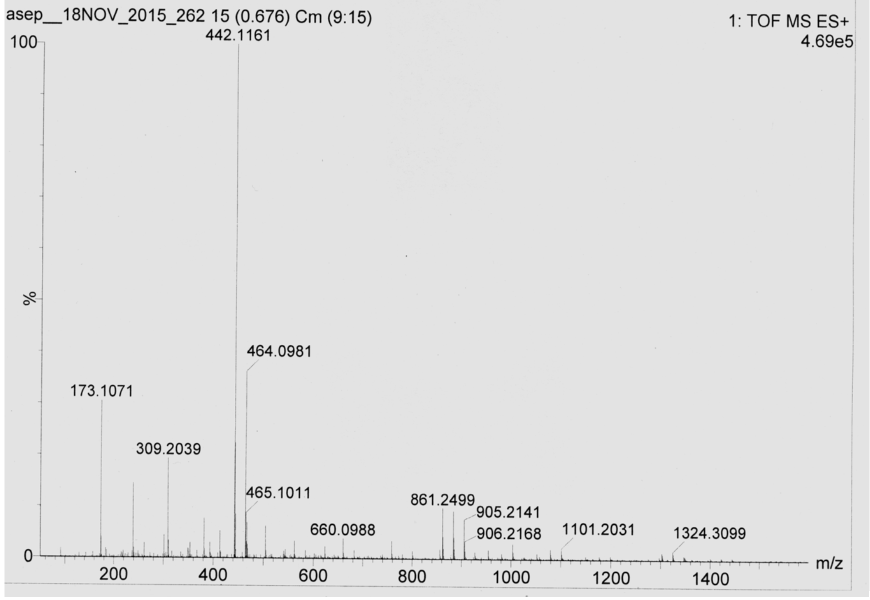

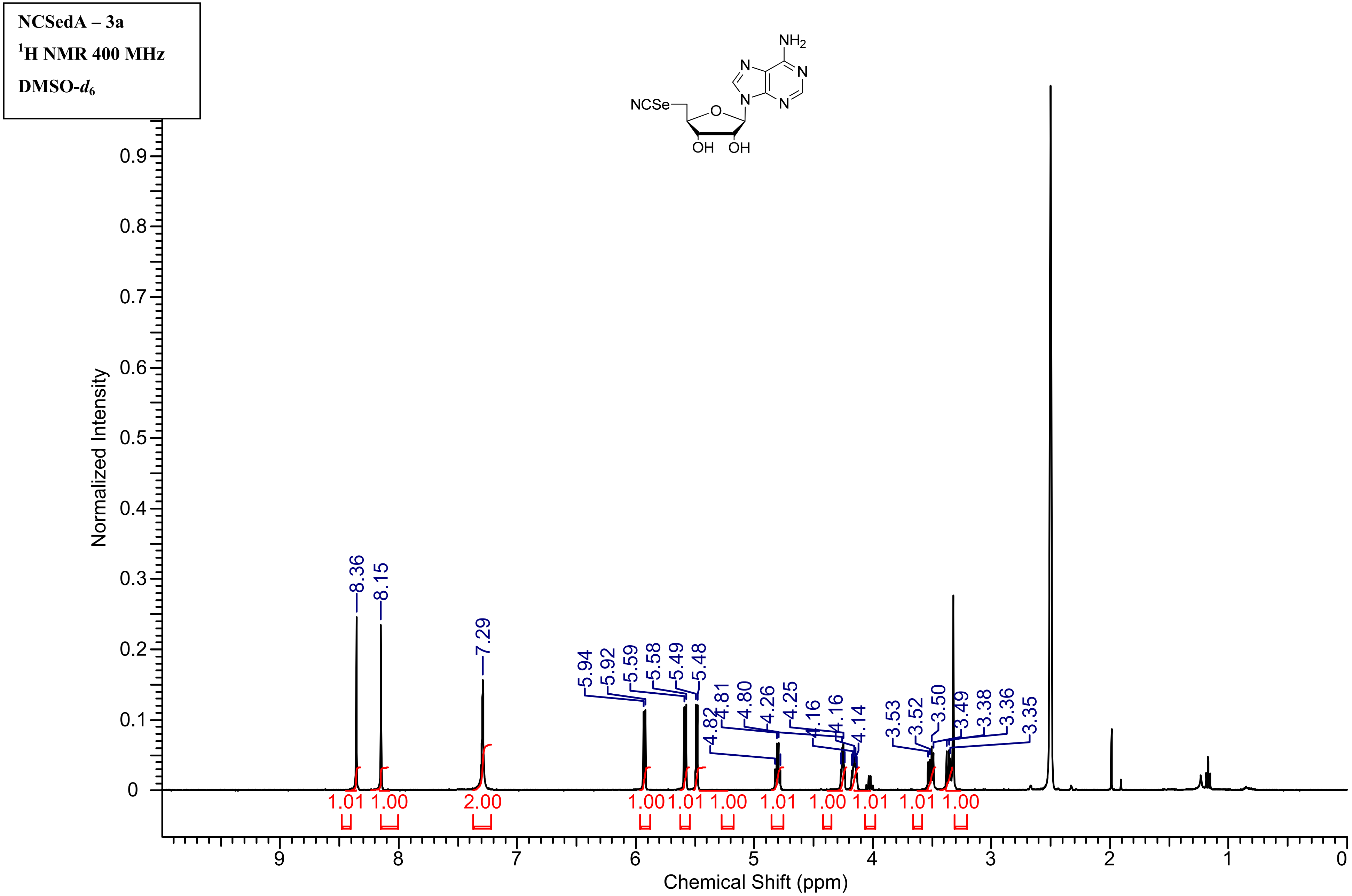
NCSedA – 3a
1H NMR 400 MHz
DMSO-d6
NH2
N
N
N
N
NCSe
O
OH
OH
8.36
8.15
7.29
5.94
5.92
5.59
5.58
5.49
5.48
4.82
4.81
4.80
4.26
4.25
4.16
4.16
4.14
3.53
3.52
3.50
3.49
3.38
3.36
3.35
1.01
1.00
2.00
1.00
1.01
1.00
1.01
1.00
1.01
1.01
1.00
Normalized Intensity
Chemical Shift (ppm)

NCSedA – 3a
^{13}C NMR 151 MHz
DMSO-d_6

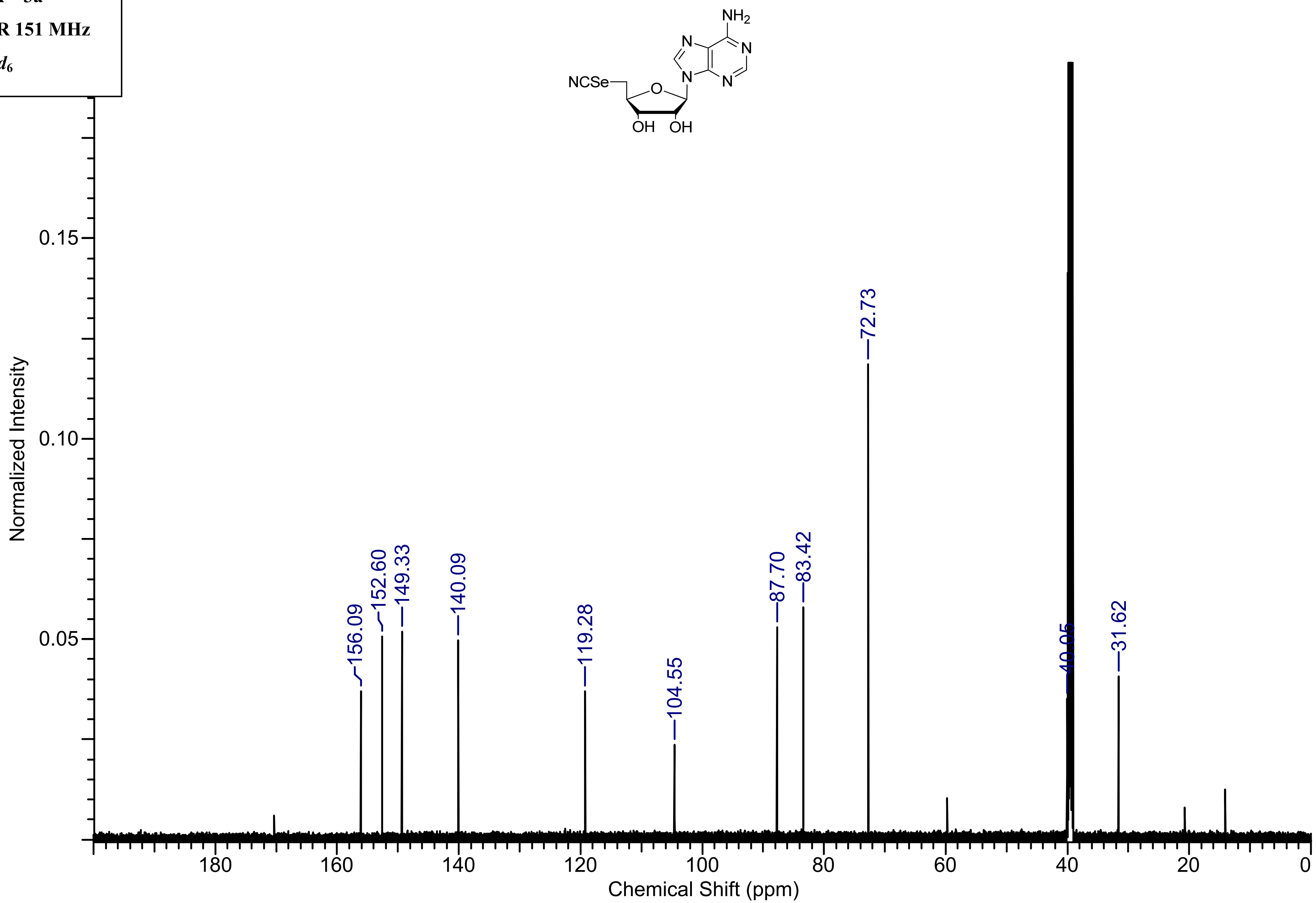

NCSedA – 3a

DEPT NMR 151 MHz

DMSO-d_6

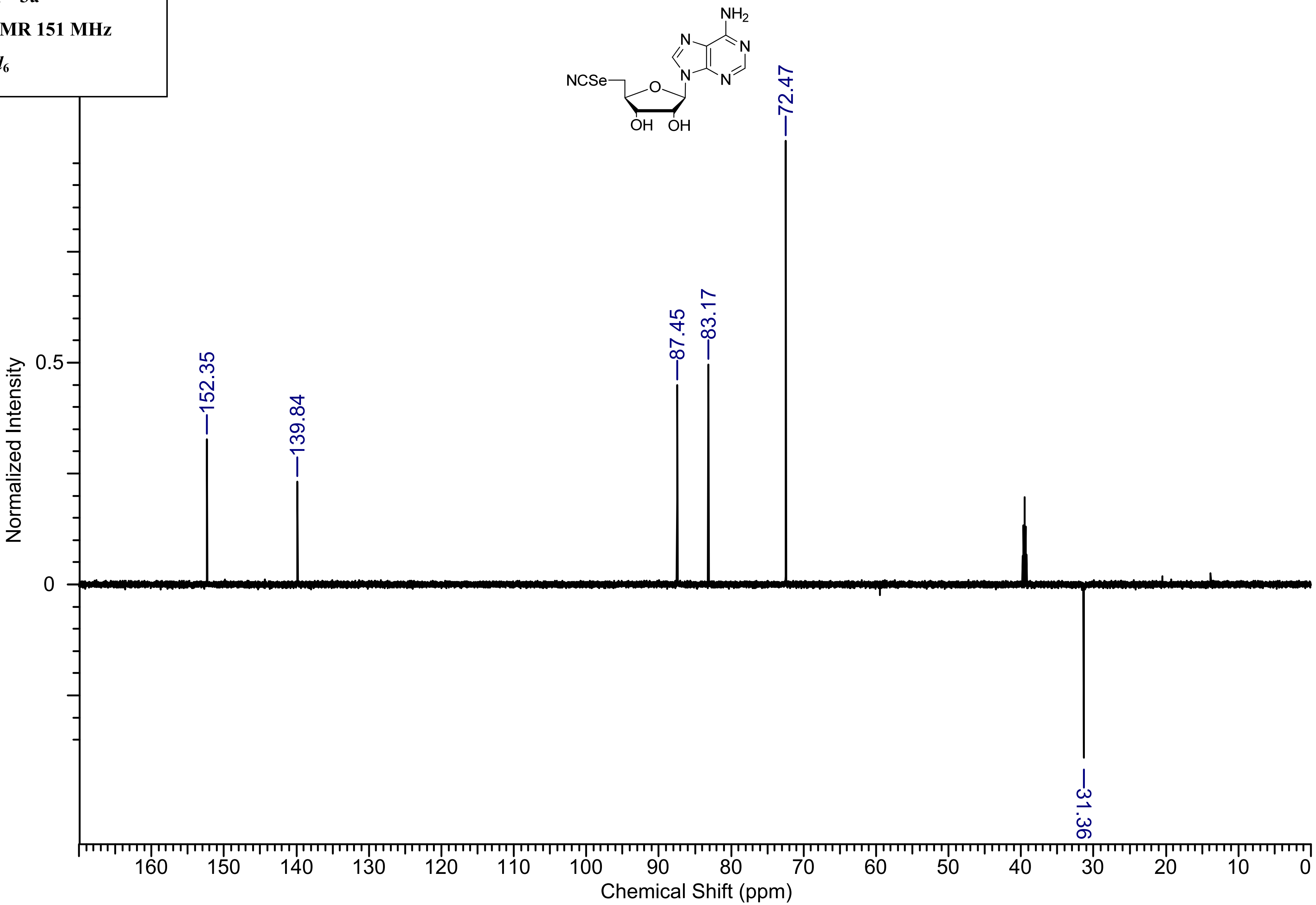

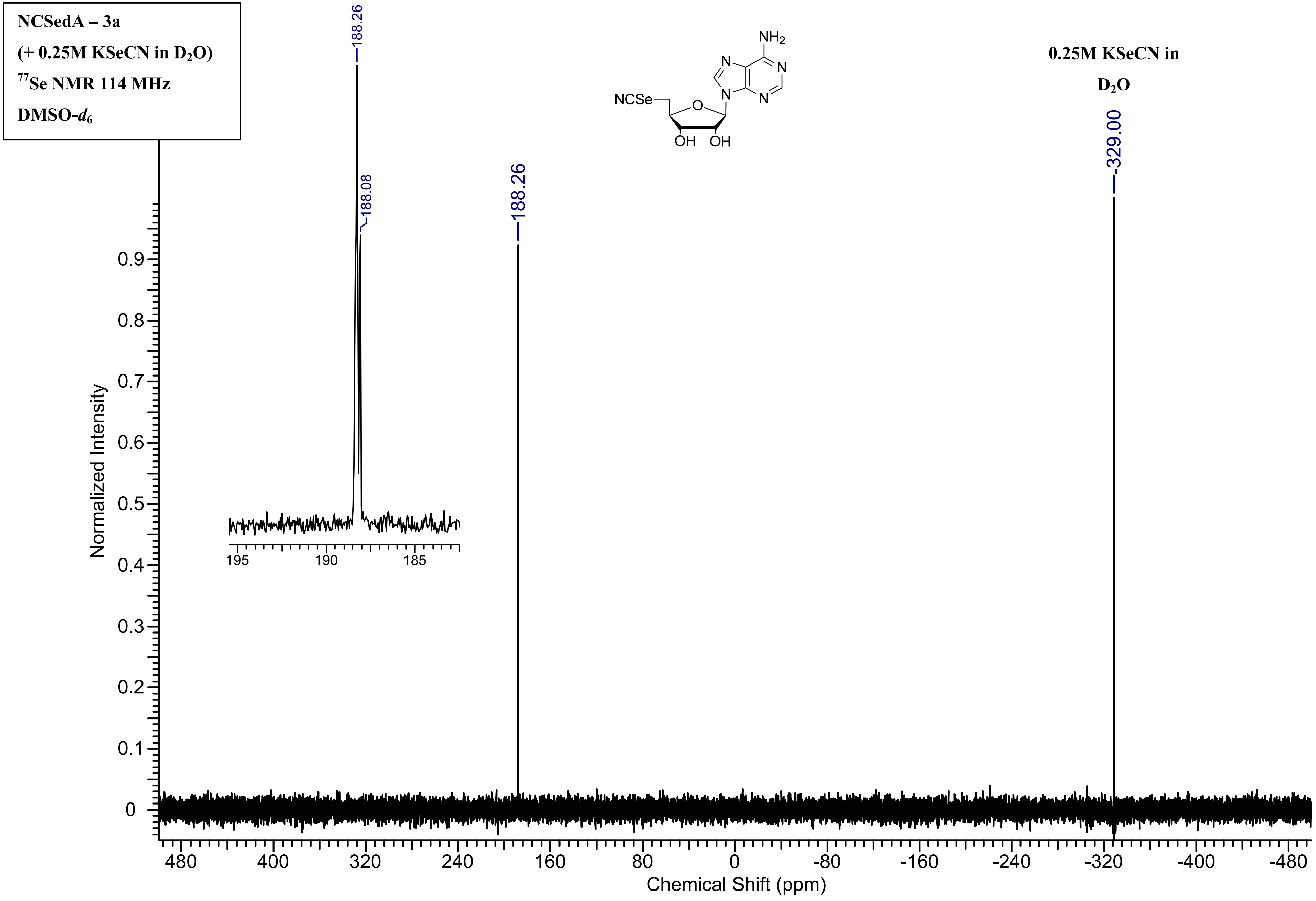
NCSedA – 3a
(+ 0.25M KSeCN in D2O)
77Se NMR 114 MHz
DMSO-d6
188.26
188.08
195
190
185
188.26
NH2
N
N
N
N
NCSe
O
OH
OH
0.25M KSeCN in
D2O
-329.00
Normalized Intensity
0.9
0.8
0.7
0.6
0.5
0.4
0.3
0.2
0.1
0
480
400
320
240
160
80
0
-80
-160
-240
-320
-400
-480
Chemical Shift (ppm)

NCSedA – 3a

HRMS (ESI, positive ion)

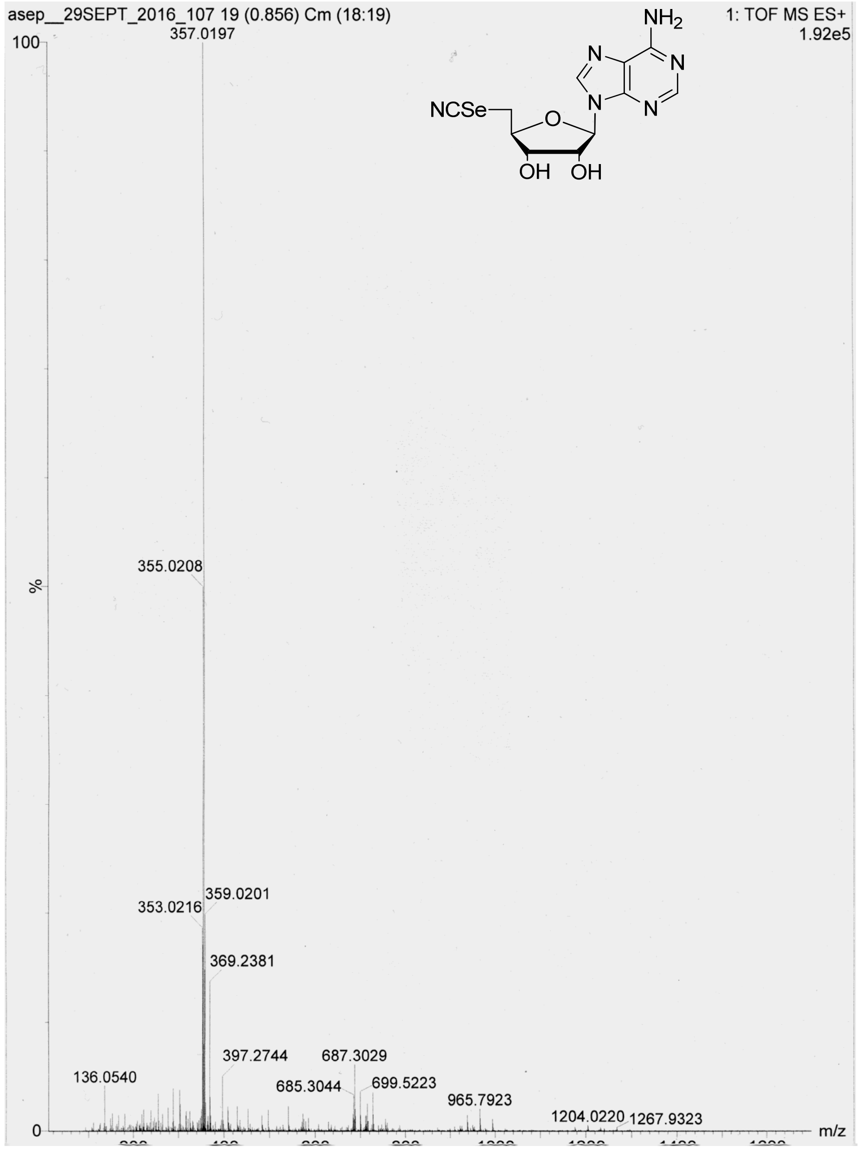

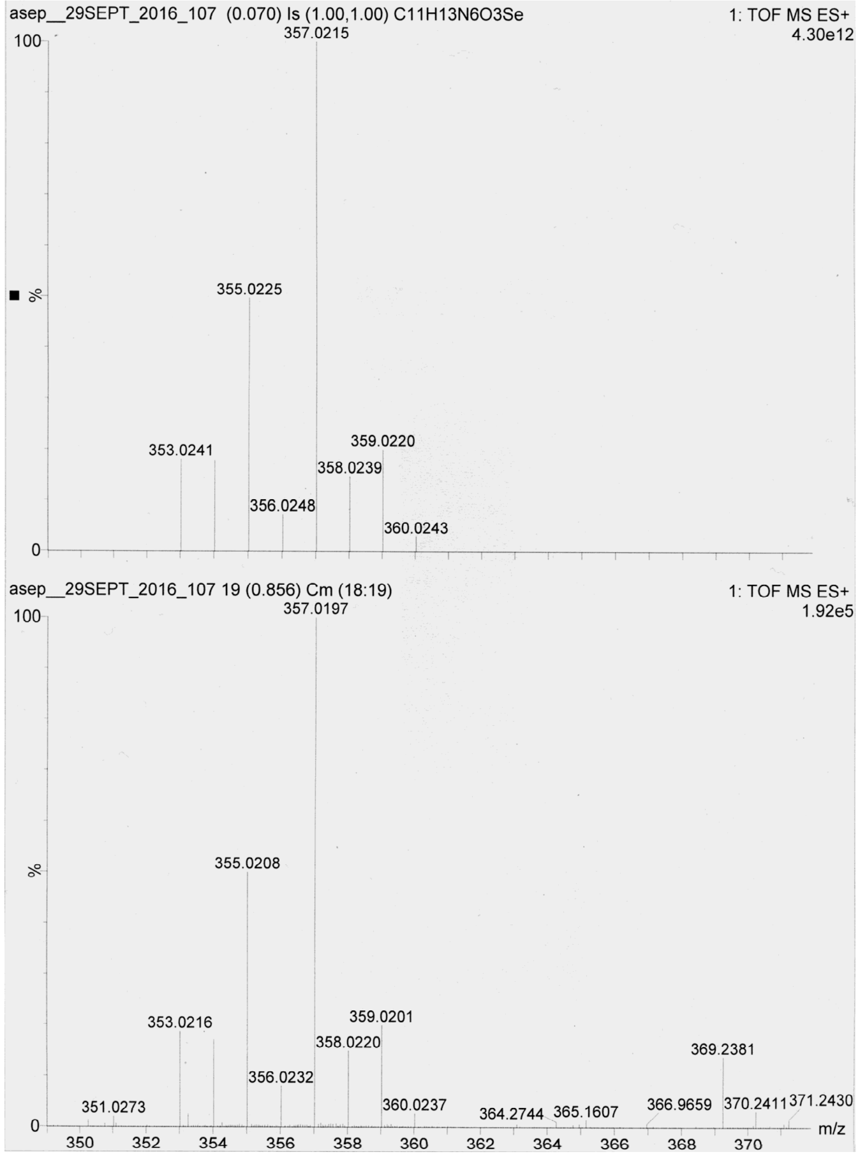

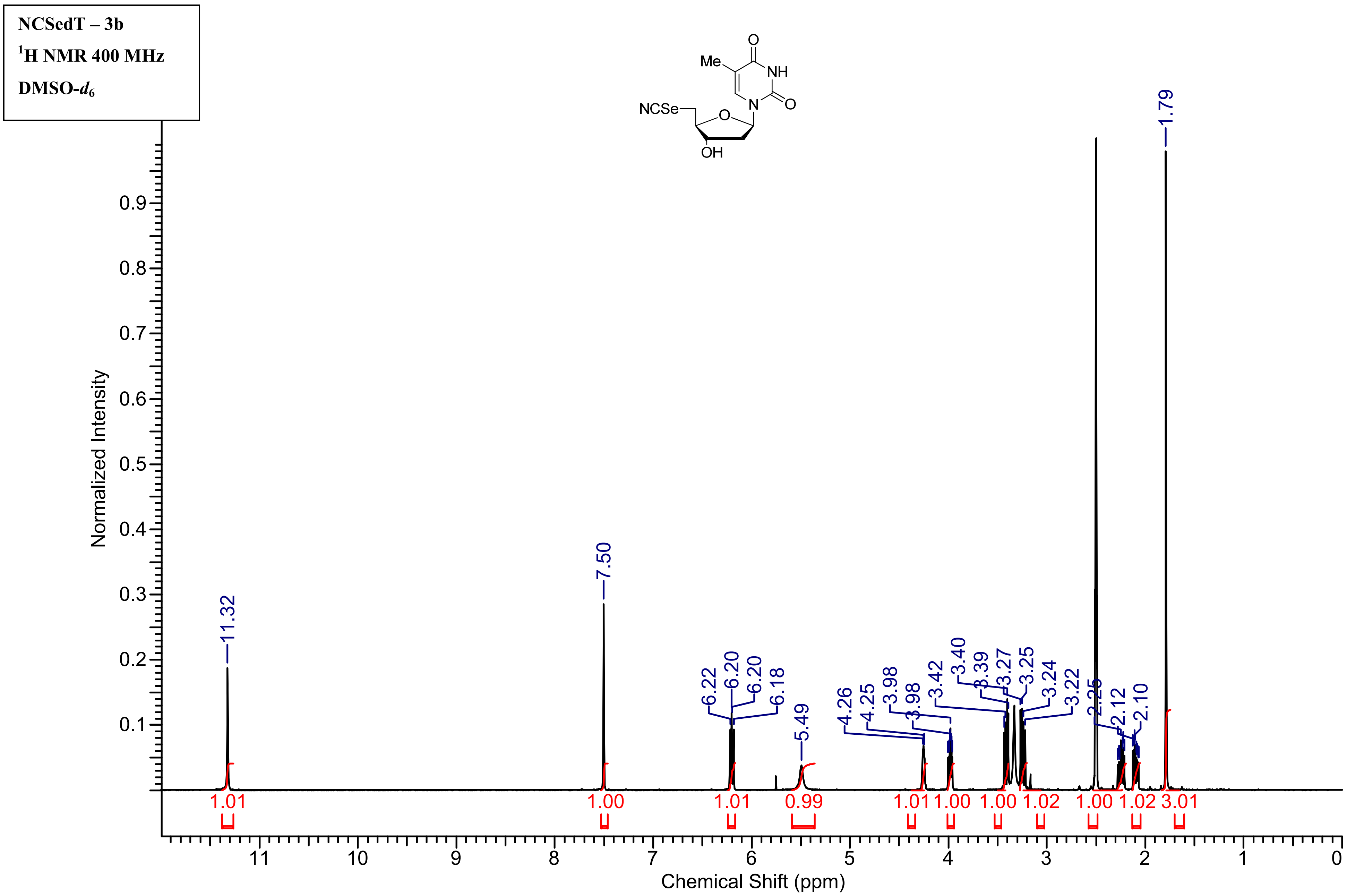
NCSedT – 3b
1H NMR 400 MHz
DMSO-d6
Me
NH
O
NCSe
N
OH
Normalized Intensity
Chemical Shift (ppm)
11.32
7.50
6.22
6.20
6.20
6.18
5.49
4.26
4.25
3.98
3.98
3.42
3.40
3.39
3.27
3.25
3.24
3.22
2.25
2.12
2.10
1.79
1.01
1.00
1.01
0.99
1.01
1.00
1.00
1.02
1.00
1.02
3.01

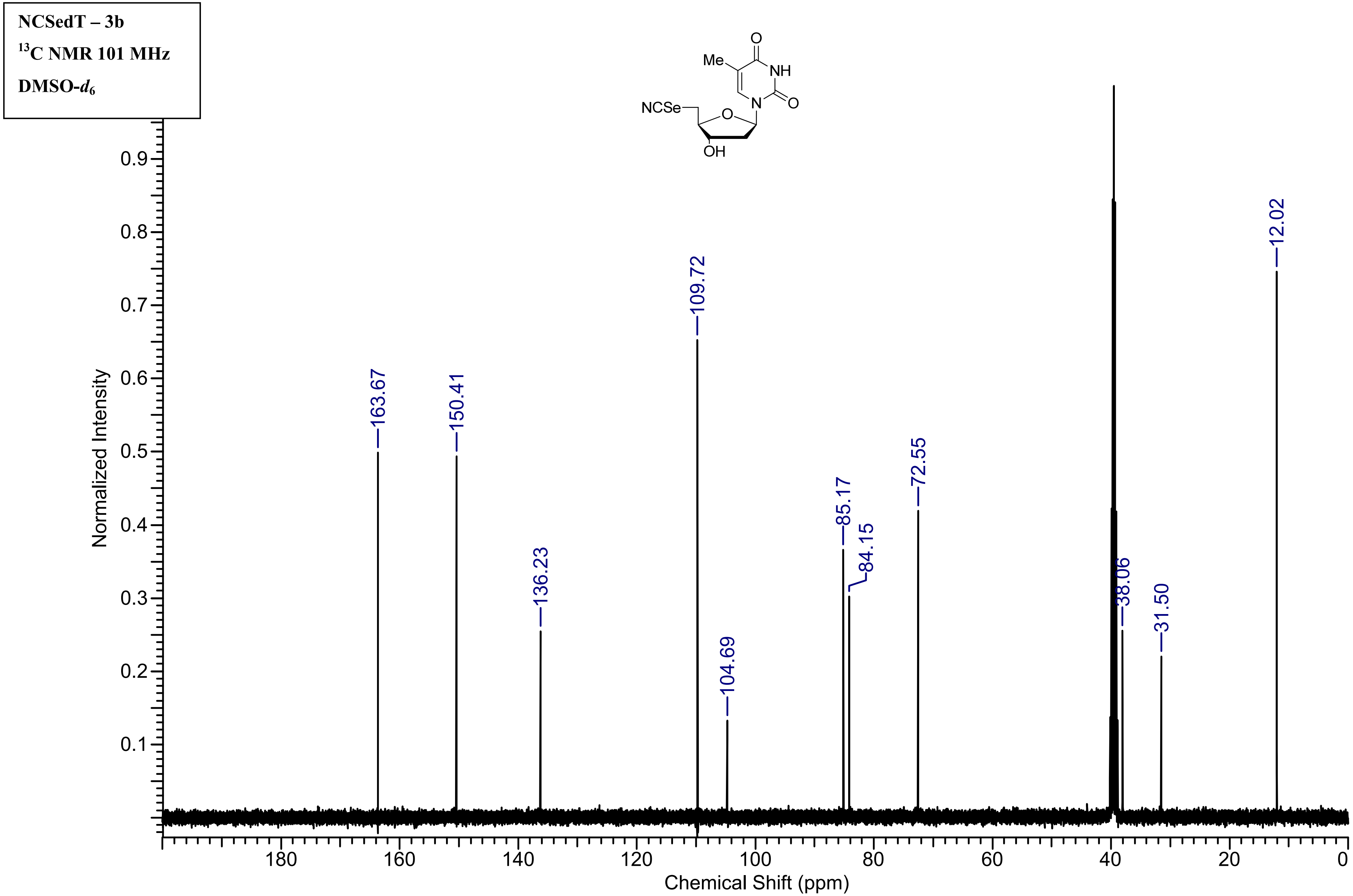
NCSedT – 3b
13C NMR 101 MHz
DMSO-d6
Me
O
NH
N
O
NCSe
O
OH
163.67
150.41
136.23
109.72
104.69
85.17
84.15
72.55
38.06
31.50
12.02
Normalized Intensity
Chemical Shift (ppm)

NCSedT – 3b
DEPTQ NMR 101 MHz
DMSO-d_6

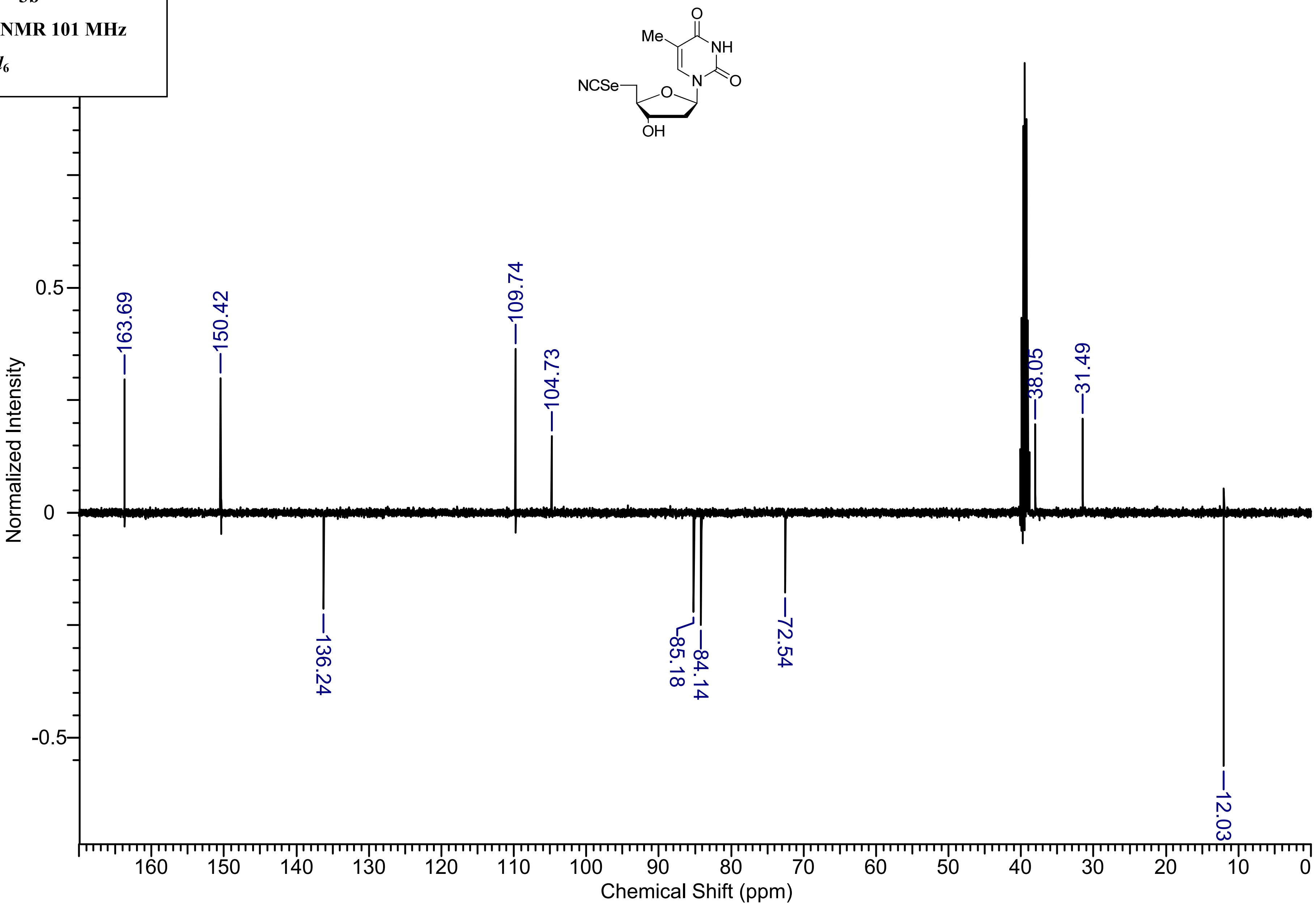

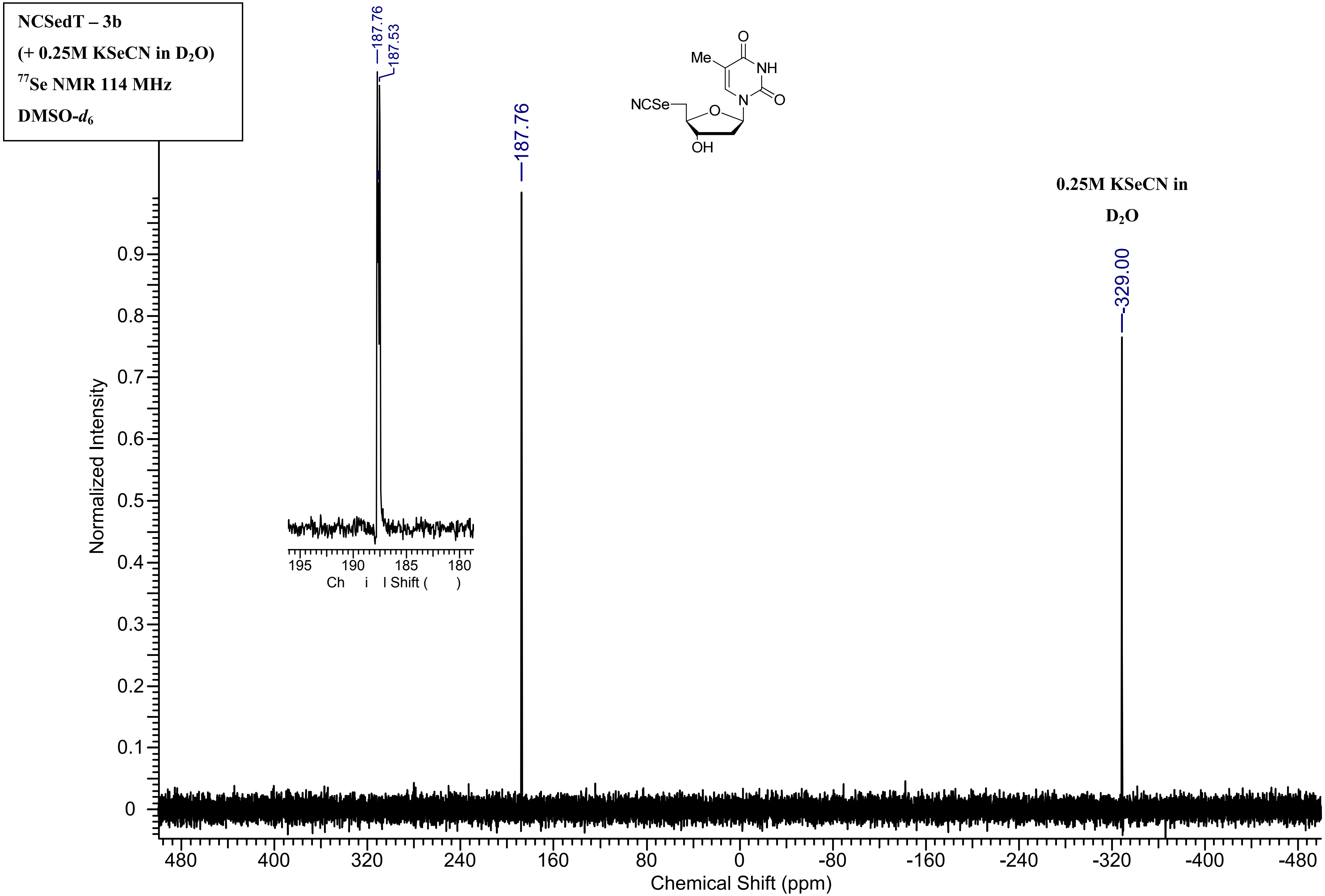
NCSedT – 3b
(+ 0.25M KSeCN in D2O)
77Se NMR 114 MHz
DMSO-d6
187.76
187.53
187.76
0.25M KSeCN in D2O
-329.00
Me
NH
O
N
NCSe
OH
Normalized Intensity
Chemical Shift (ppm)

NCSedT – 3b

HRMS (ESI, positive ion)

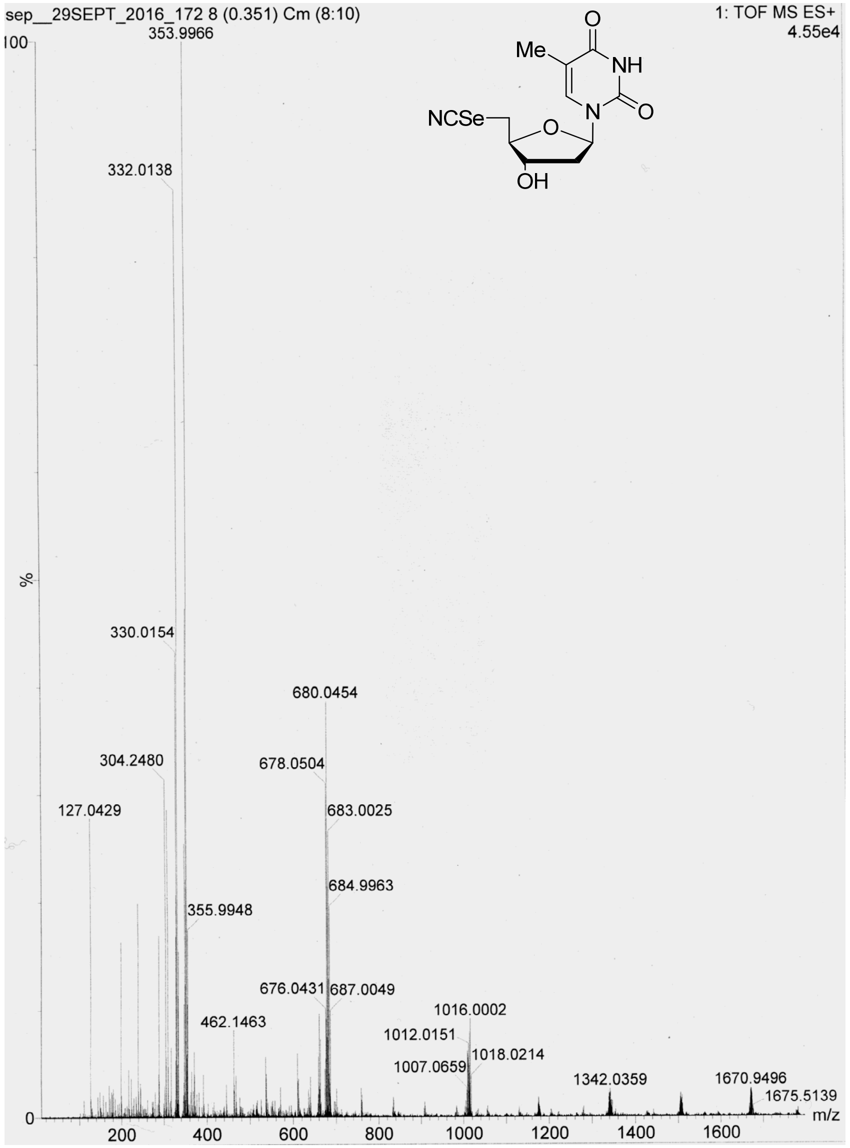

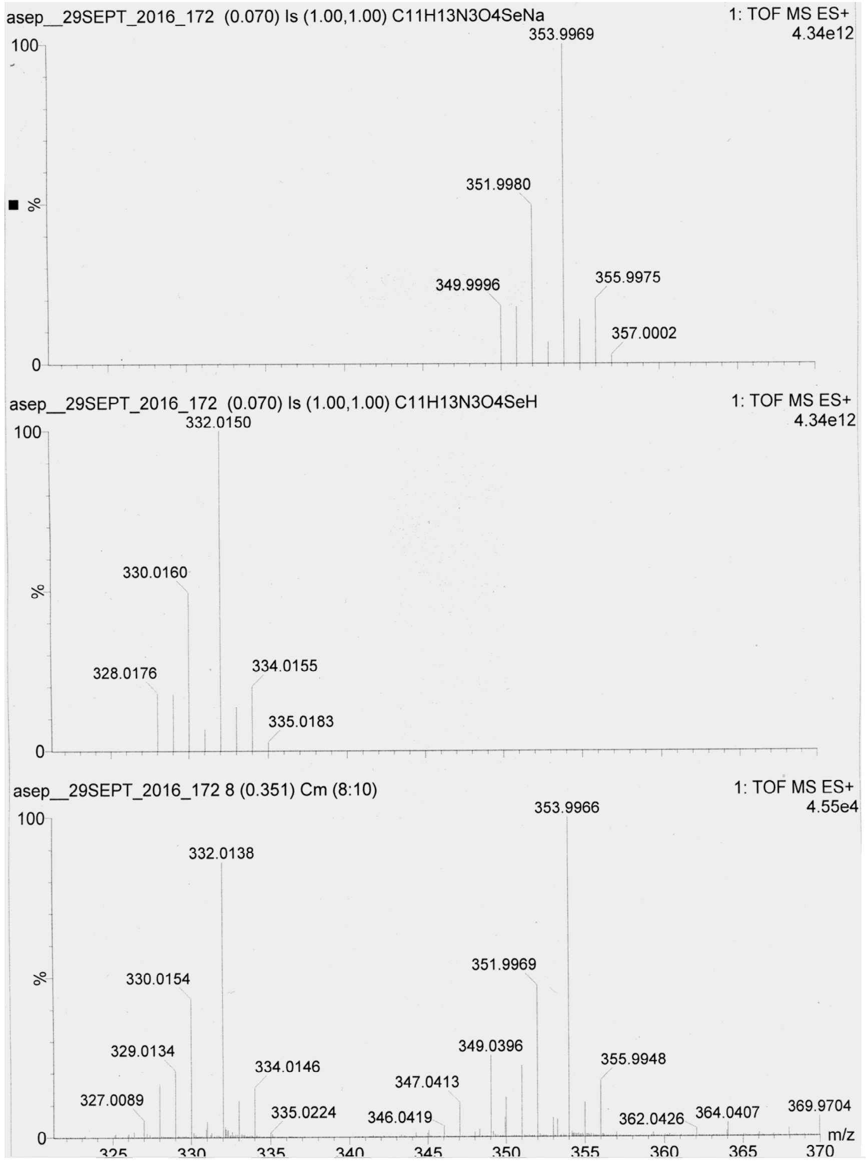

Crude NCSedG – 3c
^{1}H NMR 400 MHz
DMSO-d_6

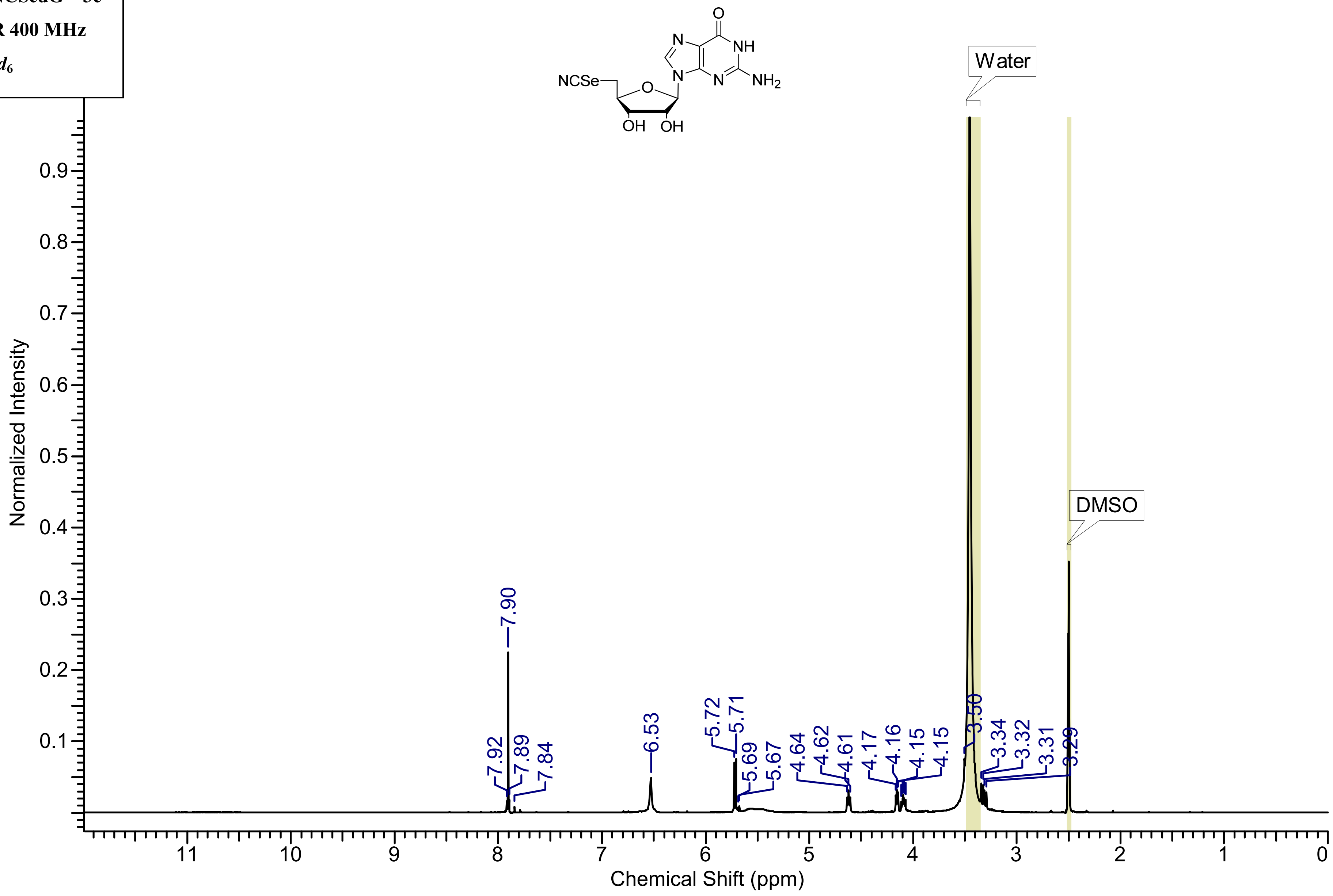

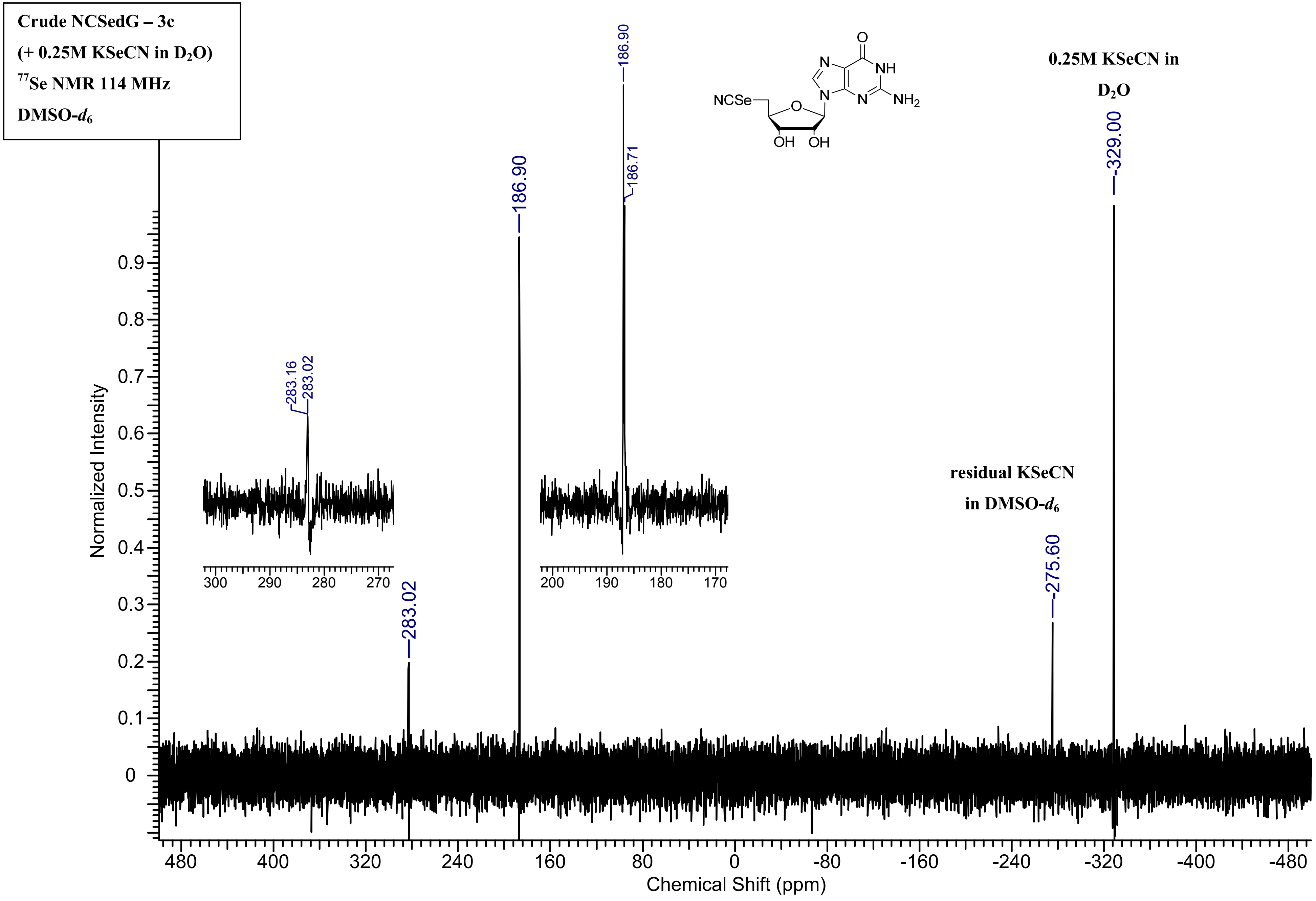

Crude NCSedG – 3c
(+ 0.25M KSeCN in D2O)
77Se NMR 114 MHz
DMSO-d6
0.25M KSeCN in D2O
residual KSeCN in DMSO-d6
NCSe
OH OH
NH
NH2
186.90
186.71
283.16
283.02
329.00
275.60
Normalized Intensity
Chemical Shift (ppm)

Crude NCSedG – 3c

HRMS (ESI, negative ion)

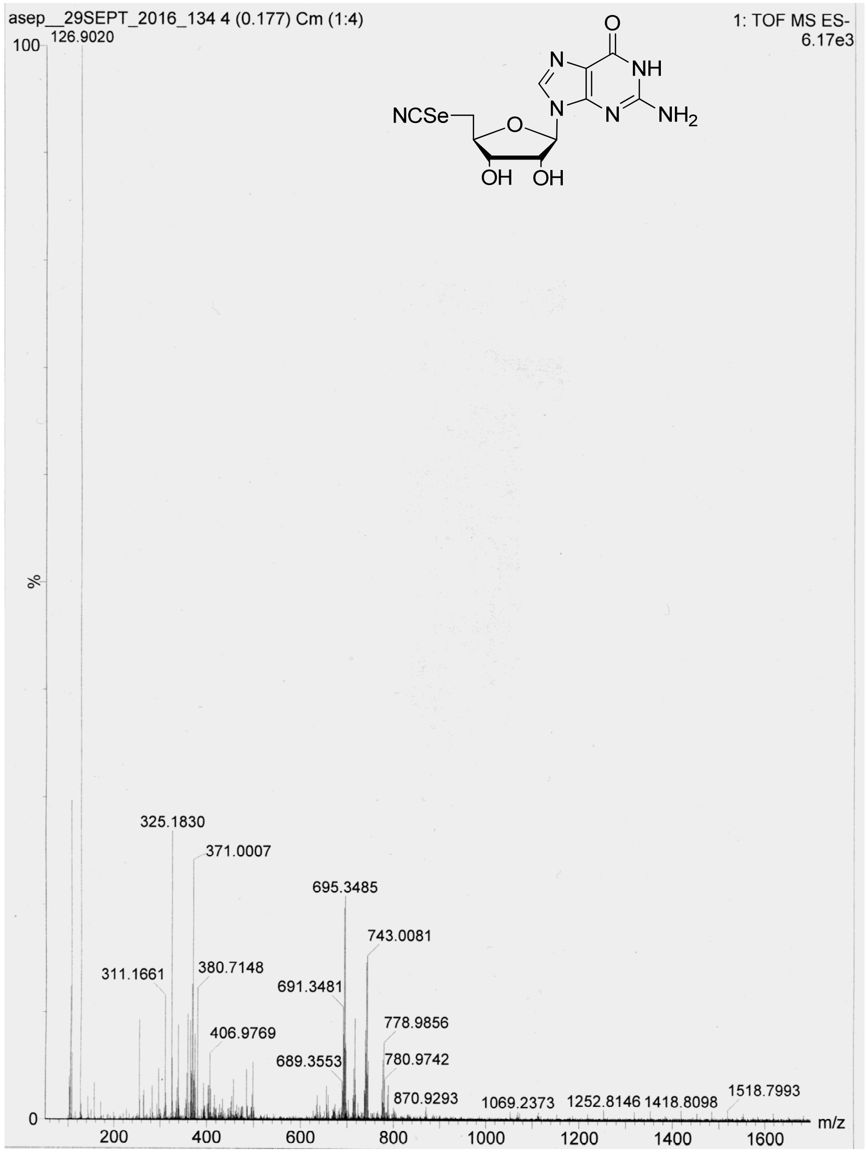

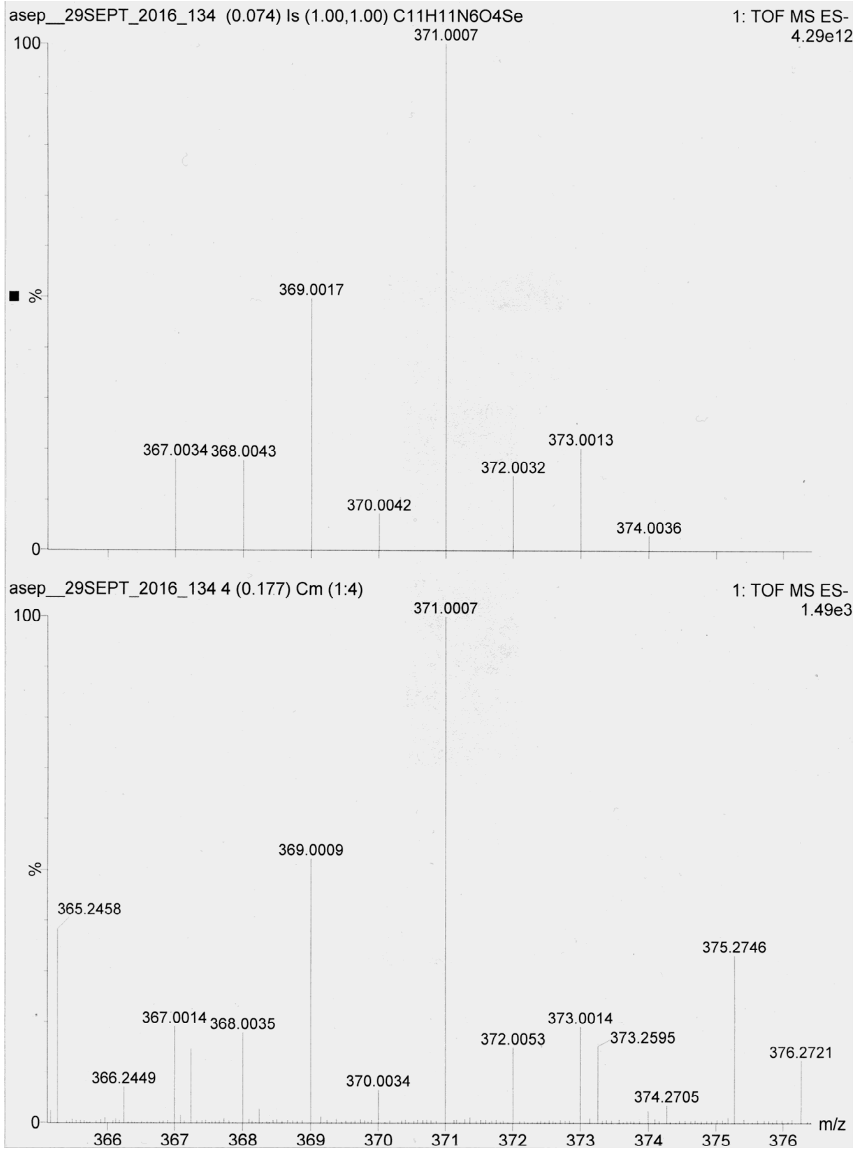

Crude NCSedG – 3c

HRMS (ESI, negative ion)

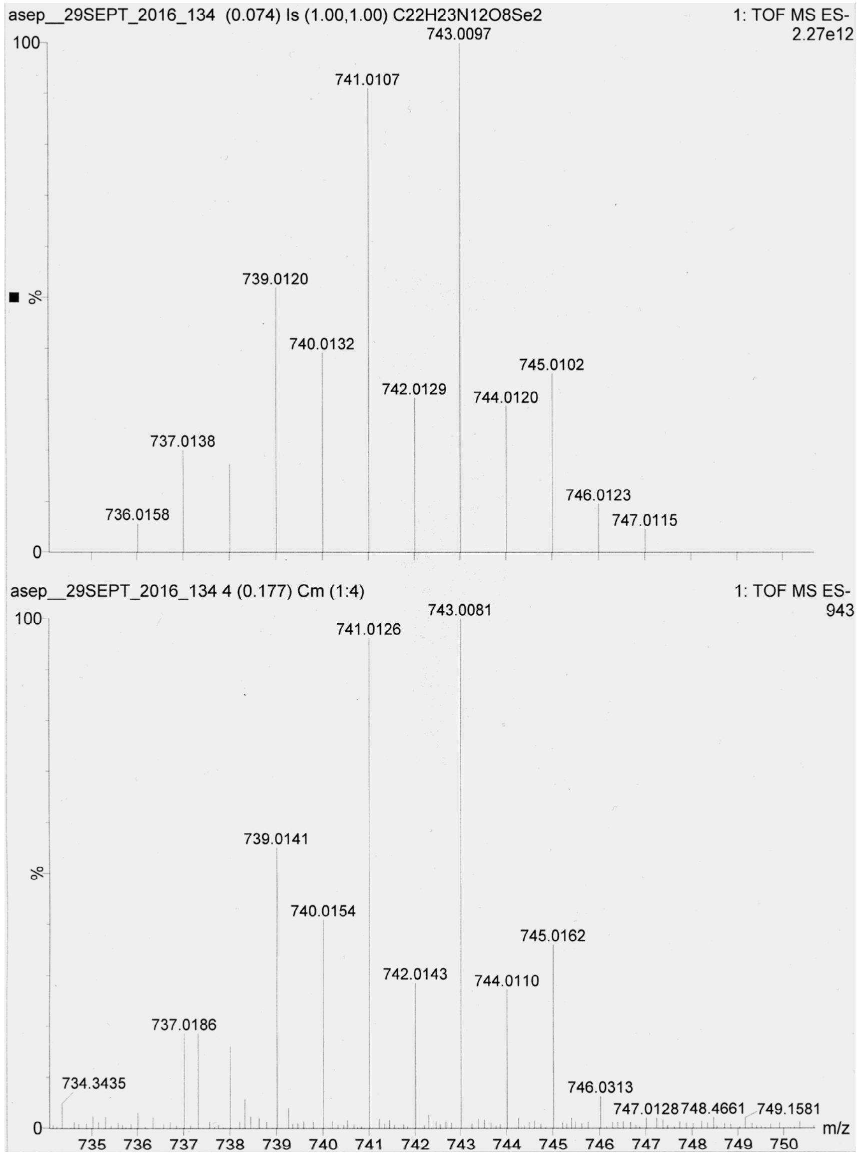

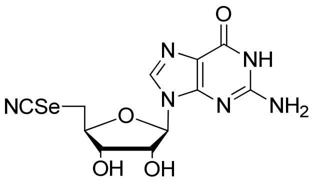

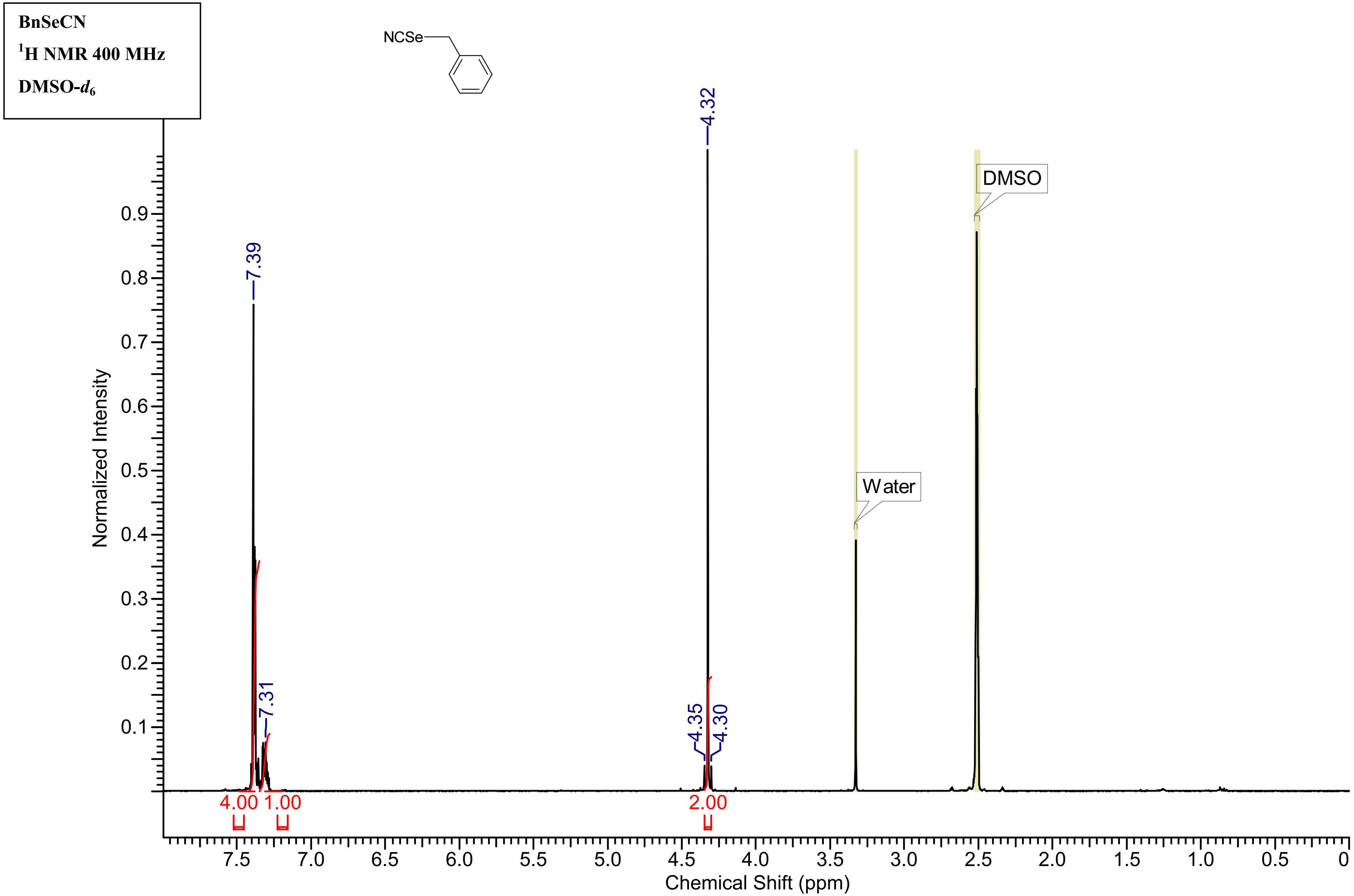
BnSeCN
^{1}H NMR 400 MHz
DMSO-d_6
NCSe
7.39
7.31
4.32
4.35
4.30
Water
DMSO
4.00
1.00
2.00
Normalized Intensity
Chemical Shift (ppm)

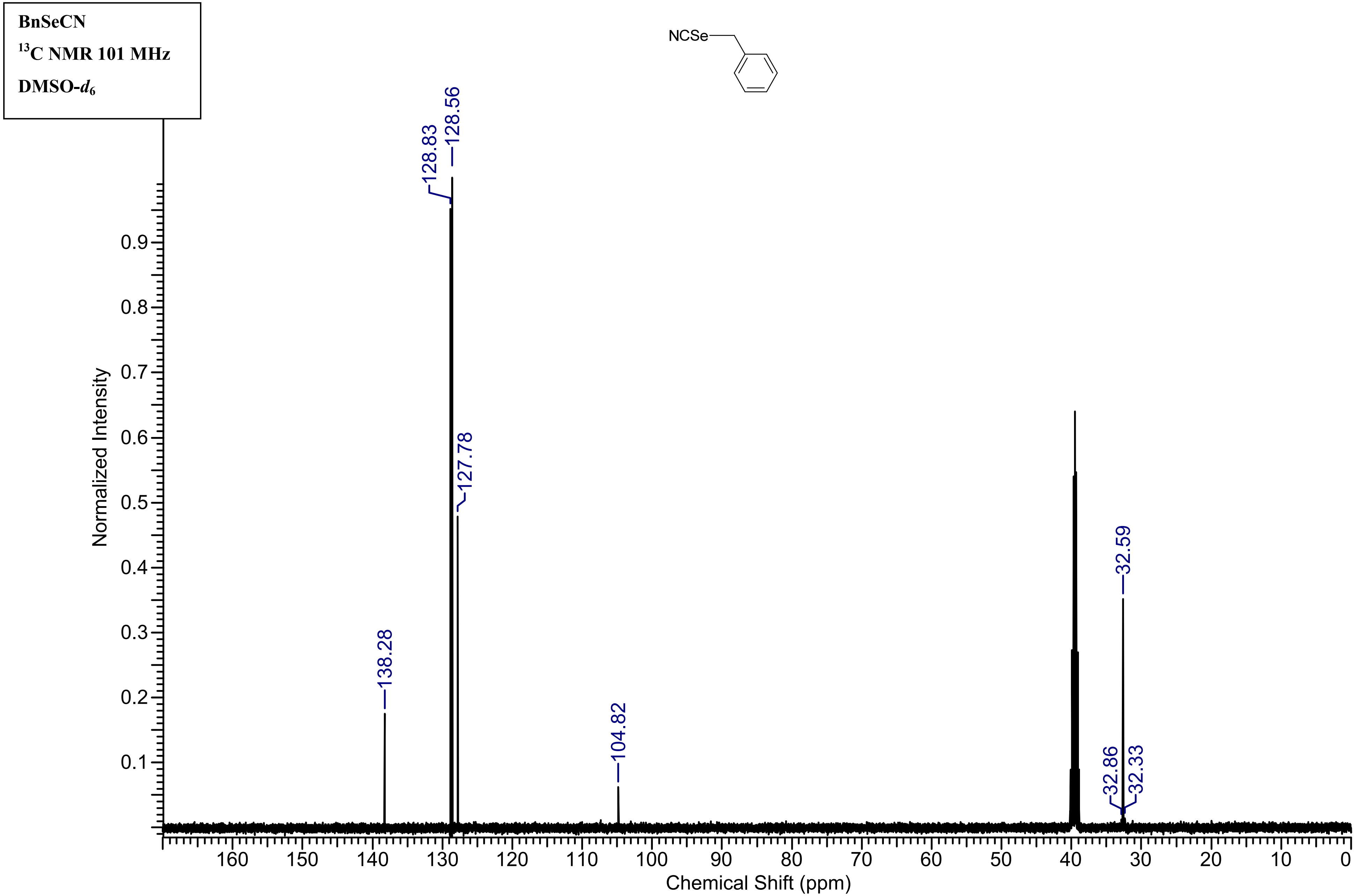
BnSeCN
13C NMR 101 MHz
DMSO-d6
NCSe
128.83
128.56
127.78
138.28
104.82
32.59
32.86
32.33
Normalized Intensity
0.9
0.8
0.7
0.6
0.5
0.4
0.3
0.2
0.1
160
150
140
130
120
110
100
90
80
70
60
50
40
30
20
10
0
Chemical Shift (ppm)

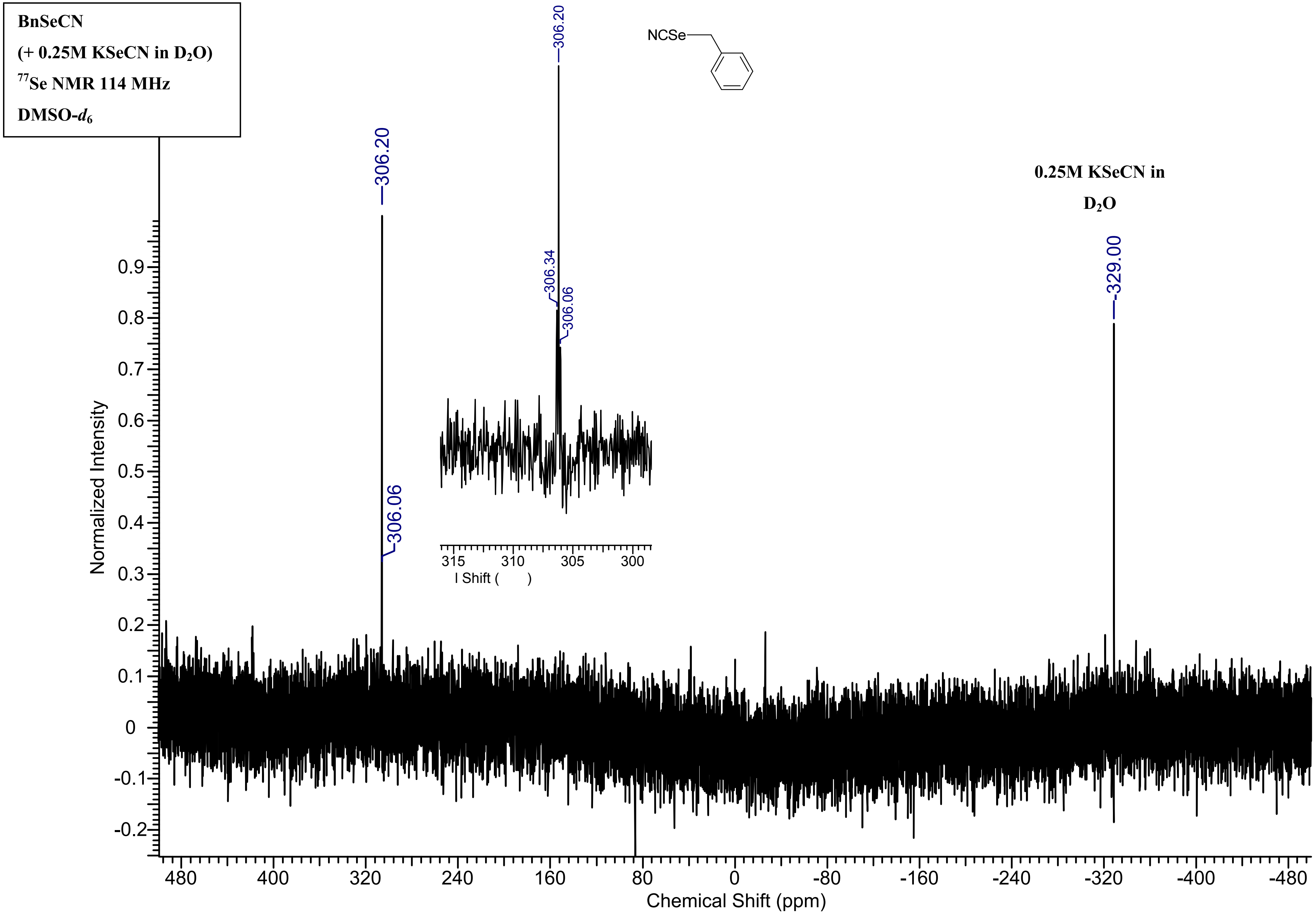
BnSeCN
(+ 0.25M KSeCN in D2O)
77Se NMR 114 MHz
DMSO-d6
NCSe
306.20
306.34
306.06
0.25M KSeCN in D2O
-329.00
Normalized Intensity
Chemical Shift (ppm)

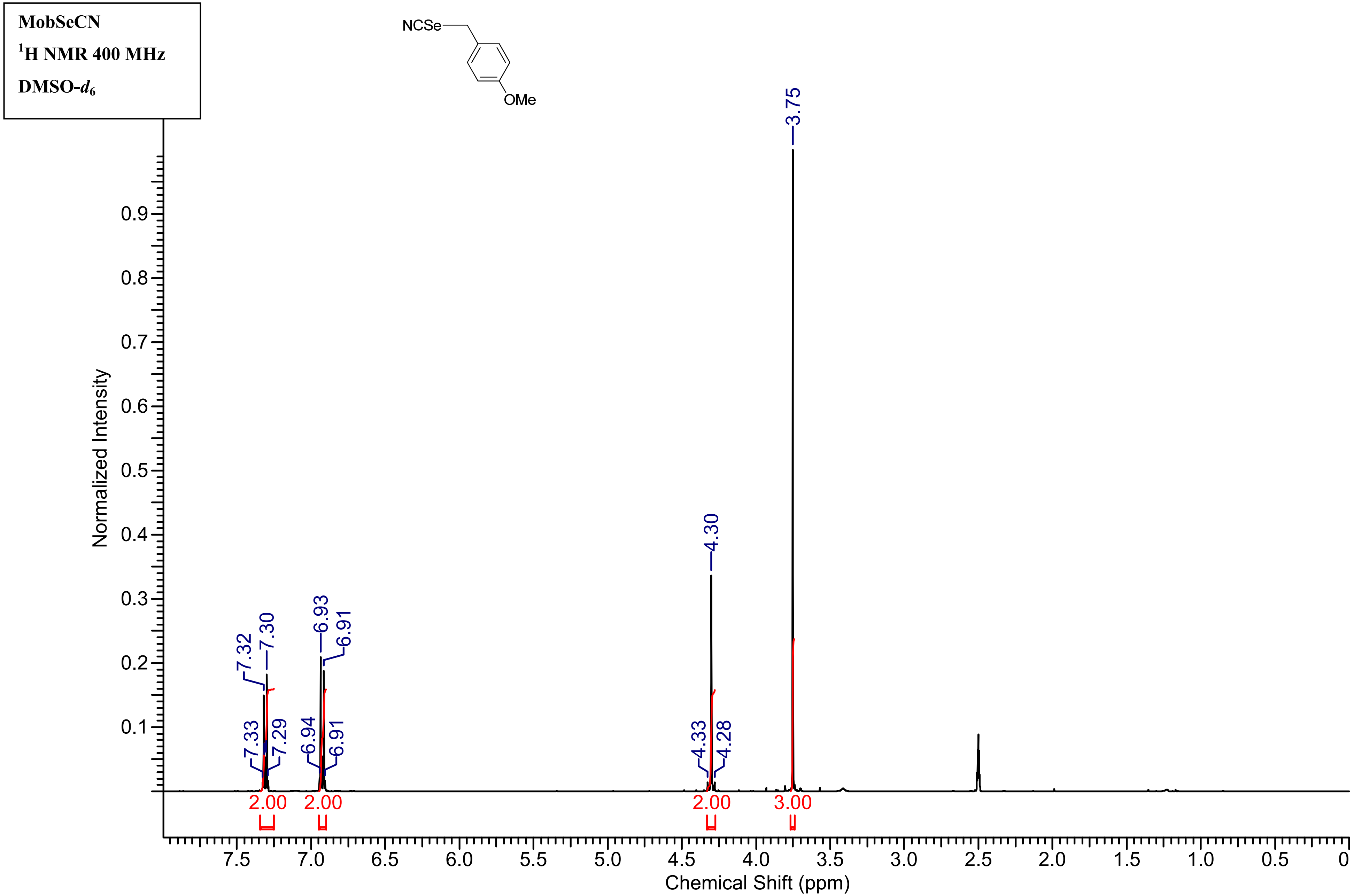
MobSeCN
1H NMR 400 MHz
DMSO-d6
NCSe
OMe
3.75
4.30
4.33
4.28
7.32
7.30
7.33
7.29
6.93
6.91
6.94
6.91
2.00
2.00
2.00
3.00
Normalized Intensity
Chemical Shift (ppm)

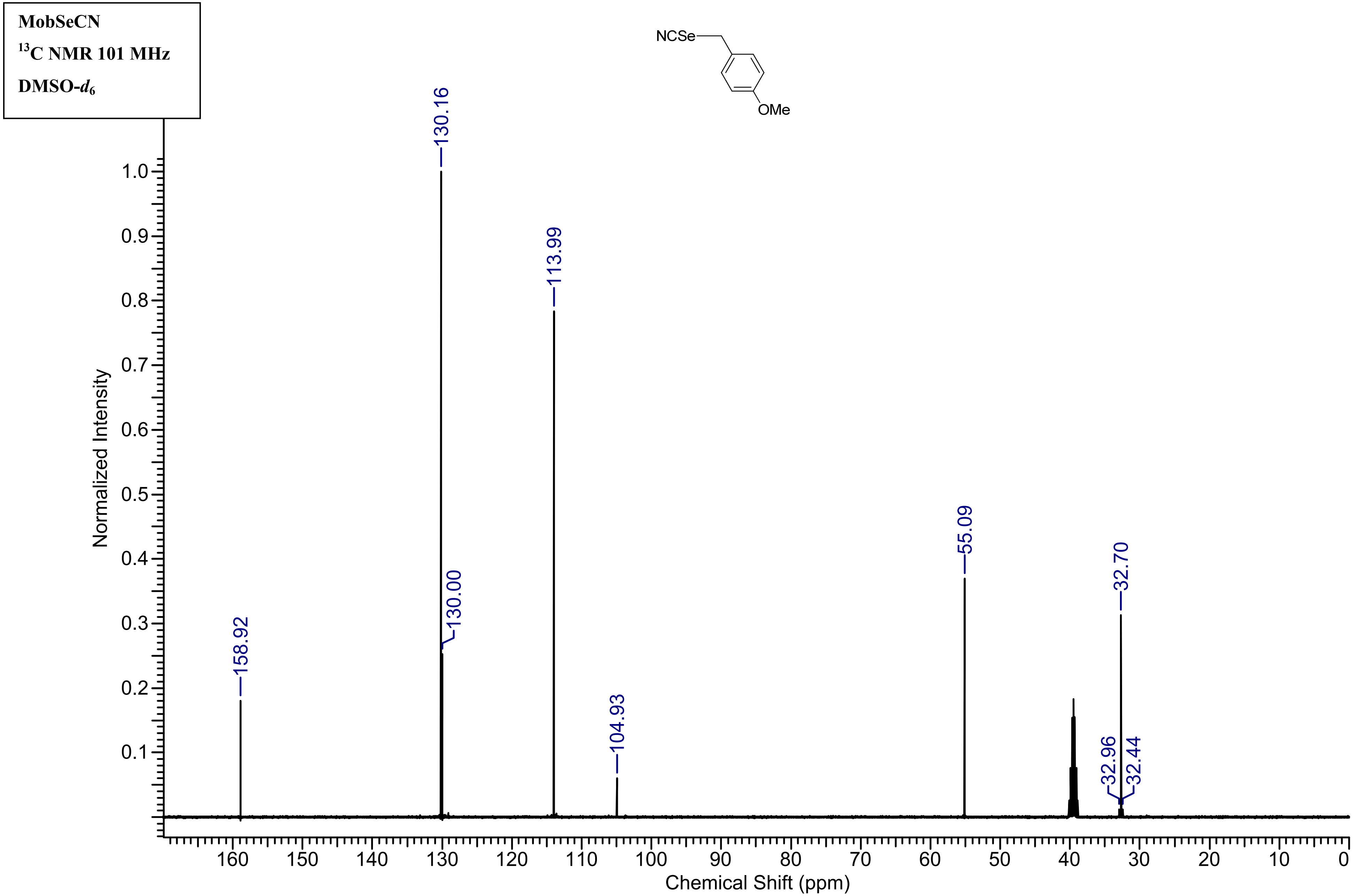
MobSeCN
13C NMR 101 MHz
DMSO-d6
NCSe
OMe
130.16
113.99
55.09
32.70
158.92
130.00
104.93
32.96
32.44
Normalized Intensity
Chemical Shift (ppm)
1.0
0.9
0.8
0.7
0.6
0.5
0.4
0.3
0.2
0.1
160
150
140
130
120
110
100
90
80
70
60
50
40
30
20
10
0

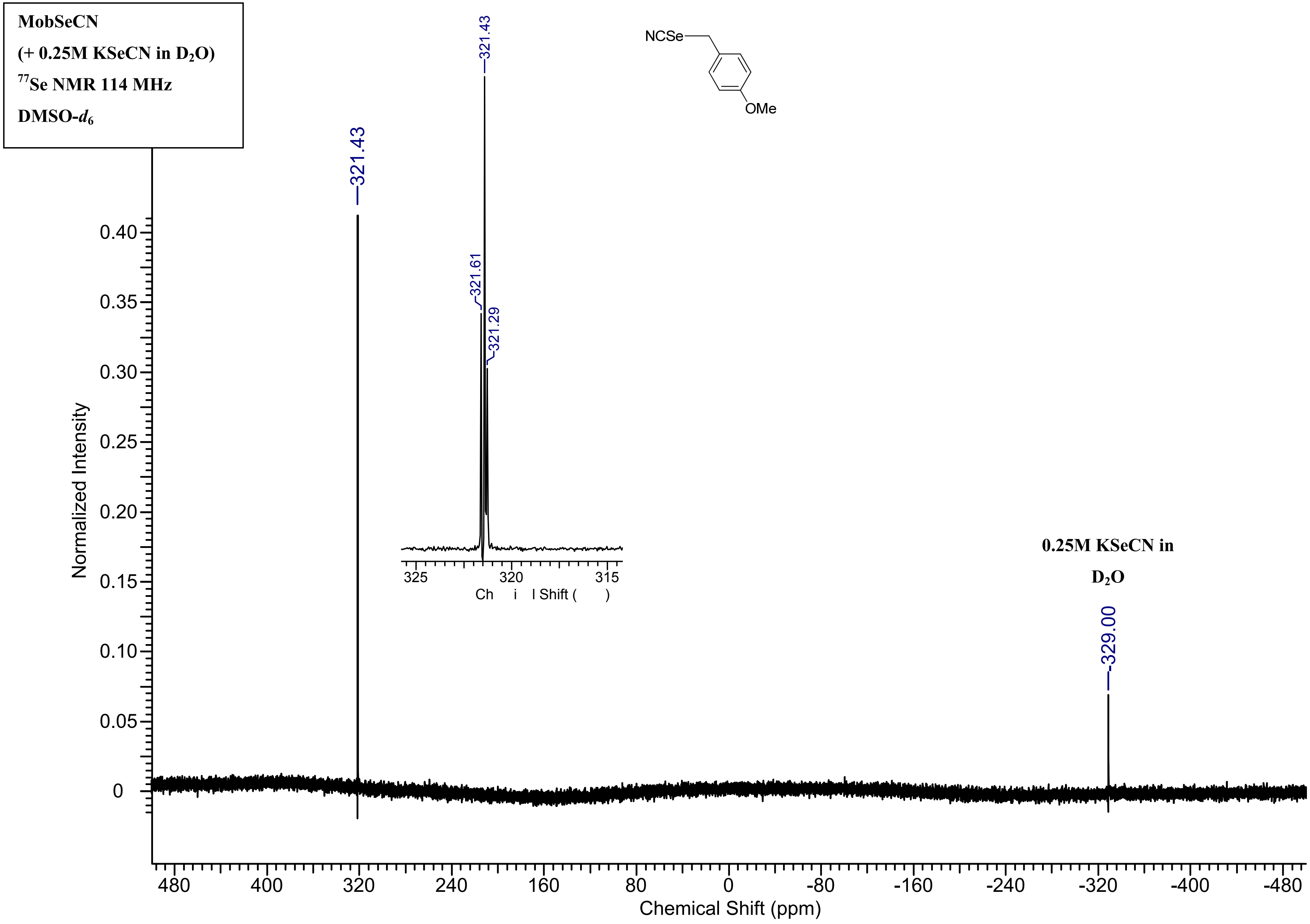
MobSeCN
(+ 0.25M KSeCN in D2O)
77Se NMR 114 MHz
DMSO-d6
NCSe
OMe
321.43
321.61
321.29
329.00
0.25M KSeCN in D2O
Normalized Intensity
Chemical Shift (ppm)
